# The incorporation of acetylated LAP-TGF-β1 proteins into exosomes promotes TNBC cell dissemination in lung micro-metastasis

**DOI:** 10.1186/s12943-024-01995-z

**Published:** 2024-04-25

**Authors:** Pei Yu, Yubao Han, Lulu Meng, Zengying Tang, Zhiwei Jin, Zhenzhen Zhang, Yunjiang Zhou, Jun Luo, Jianguang Luo, Chao Han, Chao Zhang, Lingyi Kong

**Affiliations:** 1 State Key Laboratory of Natural Medicines and Jiangsu Key Laboratory of Bioactive Natural Product Research, School of Traditional Chinese Pharmacy, China Pharmaceutical University, Nanjing, 211198 China; 2 Institute of Veterinary Science, Jiangsu Academy of Agricultural Sciences, Nanjing, 210014 China; 3 State Key Laboratory of Natural Medicines, School of Basic Medicine and Clinical Pharmacy, China Pharmaceutical University, Nanjing, 211198 China

## Abstract

Triple-negative breast cancer (TNBC) stands as the breast cancer subtype with the highest recurrence and mortality rates, with the lungs being the common site of metastasis. The pulmonary microenvironment plays a pivotal role in the colonization of disseminated tumor cells. Herein, this study highlights the crucial role of exosomal LAP-TGF-β1, the principal form of exosomal TGF-β1, in reshaping the pulmonary vascular niche, thereby facilitating TNBC lung metastasis. Although various strategies have been developed to block TGF-β signaling and have advanced clinically, their significant side effects have limited their therapeutic application. This study demonstrates that in lung metastatic sites, LAP-TGF-β1 within exosomes can remarkably reconfigure the pulmonary vascular niche at lower doses, bolstering the extravasation and colonization of TNBC cells in the lungs. Mechanistically, under the aegis of the acetyltransferase TIP60, a non-canonical KFERQ-like sequence in LAP-TGF-β1 undergoes acetylation at the K304 site, promoting its interaction with HSP90A and subsequent transport into exosomes. Concurrent inhibition of both HSP90A and TIP60 significantly diminishes the exosomal burden of LAP-TGF-β1, presenting a promising therapeutic avenue for TNBC lung metastasis. This study not only offers fresh insights into the molecular underpinnings of TNBC lung metastasis but also lays a foundation for innovative therapeutic strategies.

**Supplementary Information:**

The online version contains supplementary material available at 10.1186/s12943-024-01995-z.

## Introduction

Breast cancer remains the most prevalent malignancy among women, with triple-negative breast cancer (TNBC) representing the most aggressive subtype, notorious for its high recurrence and mortality rates [1]. The lungs serve as a primary metastatic site for TNBC, acting as the first major capillary bed encountered by TNBC cells that escape into the bloodstream. While metastasis signifies the terminal and most lethal phase of malignant tumor progression, trans-endothelial migration is fundamental to tumor cell dissemination [2, 3]. Yet, a mere 0.01% of cells that migrate from the primary tumor succeed in extravasating at distant secondary sites [4, 5]. Emerging evidence posits that metastasis can undergo multiple rounds of reseeding, often transpiring through spread between distal sites rather than direct migration from the primary tumor [6–9]. Lung micrometastatic tumors provide the “soil” by altering the vascular microenvironment for the formation of substantial lung metastatic tumors [5, 10, 11]. This microenvironment establishment appears pivotal for the colonization of disseminating tumor cells [12], highlighting the importance of identifying the drivers of this microenvironmental reshaping.

Among the myriad influencers, exosomes have emerged as essential mediators of intercellular communication, particularly in forging pre-metastatic niches [13]. The molecular cargo of tumor-derived exosomes, comprising proteins and nucleic acids, can modulate cellular behaviors. For instance, tumor exosomal miR-1247-3p has been shown to activate cancer-associated fibroblasts, promoting lung metastasis of liver cancer [14], and lung cancer-derived exosomal TRIM59 regulates macrophage NLRP3 inflammasome activation, fostering lung metastasis [15]. Despite the recognized significance of exosomes, the specifics of how their cargo impacts the vascular microenvironment and the subsequent facilitation of substantial metastatic tumor formation in the lungs remain elusive [16, 17]. This knowledge gap hampers the development of effective therapeutic interventions.

Tumor-derived exosomal TGF-β1, known for its roles in tumor resistance, immune evasion, and epithelial-mesenchymal transition (EMT), has been extensively documented [18–22]. The inactive form of this molecule, LAP-TGF-β1, is a protein dimer composed of latency-associated peptide (LAP) and the TGF-β1 active subunit [23]. Activation of free LAP-TGF-β1 hinges on cell-specific integrins, proteases, the transmembrane protein GARP, and Thrombospondin-1. Exosomal LAP-TGF-β1 boasts a unique activation pathway, wherein, post-uptake, the active TGF-β1 is released in a pH-dependent manner within acidified endosomes, fostering a delayed signaling cascade [24, 25]. Yet, the ramifications of tumor exosomal LAP-TGF-β1 on the pulmonary vascular niche remain enigmatic. Notably, in mesotheliomas and prostate cancers, LAP-TGF-β1 constitutes over 90% of exosomal TGF-β1 [26], while in normal cells, such as adipocytes, it accounts for merely 60% [24]. This disparity suggests a tumor-specific pathway for loading LAP-TGF-β1 onto exosomes.

Our investigation unveils the pivotal role of exosomal LAP-TGF-β1 in reshaping the pulmonary vascular niche during TNBC lung metastasis and its consequent contribution to substantial metastatic tumor formation. A pronounced 2.4-fold elevation of LAP-TGF-β1 in exosomes from TNBC lung metastatic lesions compared to parent breast cancer cells, and a staggering 27-fold rise when compared to normal cells such as adipocytes, was observed. Its potent effect at just 1/250th the dosage of free TGF-β1 underscores its amplified presence in fostering pulmonary vascular niche formation and driving TNBC lung metastasis progression. Silencing of exosomal LAP-TGF-β1 attenuates these exosome-driven activities, underscoring its potential as a therapeutic target in TNBC lung metastasis. Further research unveiled a non-canonical KFERQ-like sequence in the TGFB1 region of LAP-TGF-β1, wherein the K304 site undergoes acetylation mediated by acetyltransferase TIP60, facilitating interaction with HSP90A and subsequent exosomal trafficking. Concurrent inhibition of both HSP90A and TIP60 markedly diminishes exosomal LAP-TGF-β1 load, effectively curtailing TNBC lung metastasis. Given the prevalent overexpression of HSP90A and TIP60 in various tumor cells, a therapeutic strategy that jointly targets HSP90A and TIP60, aiming to diminish LAP-TGF-β1 in exosomes, could provide a novel avenue for suppressing tumor metastasis to the lungs.

## Materials and methods

Detailed procedures were provided in Supplemental Methods.

### Transfection

The sequences of LAP-TGFB1 (NM_000660.7), HSP90AA1 (NM_005348.4), TIP60 (NM_006388.4), and PCAF (NM_003884.5), as identified in NCBI, were cloned and subjected to mutations. The plasmids pcDNA3β-flag-CBP-HA (Addgene plasmid #32,908), pcDNA3.1-p300 (Addgene plasmid #23,252), and pEBB flag GCN5 (Addgene plasmid #74,784) were kindly provided by Mengshuang Fang. His-LAP-TGFB1, HA-LAP-TGFB1, HA-TGFB1, HA-LAP, HA-LAP-TGFB1 K304Q, HA-LAP-TGFB1 R303A/K304A, flag-TIP60, flag-TIP60 DN, flag-PCAF, HA-CBP, HA-p300, flag-GCN5, and His-HSP90AA1 were subcloned into the pcDNA3.1 (+) vector from General Biosystems, Inc. (Anhui, China). Specific siRNAs and a nontargeting siRNA variant were synthesized by General Biosystems (Supplementary Table 1).

For transfection procedures, both plasmids and siRNAs were introduced into 231 Parental or 231 LuT3 cells using Lipofectamine 3000, adhering strictly to the manufacturer’s protocol. At 48 h post-transfection, cells were analyzed using western blot and real-time qPCR techniques, with a subset reserved for additional experimentation.

### Exosome isolation

Exosomes were separated from cells using differential centrifugation. Cells were cultured for 48 h in DMEM supplemented with 5% exosome-depleted fetal bovine serum. The subsequent cell culture supernatant was centrifuged at 500 g for 10 min to pellet cells, followed by a spin at 2,000 g for 20 min to exclude cell debris and nonviable cells.

For in vivo extraction, 1 × 10^6^ of 231 parental, 231 LuT1, 231 LuT2, or 231 LuT3 cells were introduced into the No. 4 mammary fat pad of female NCG-HLA-A2.1 mice aged between 6 and 8 weeks. Post four weeks, blood was collected from these tumor-harboring mice after inducing deep sedation using 150 mg/kg pentobarbital. Plasma-derived exosomes were isolated with a centrifugation protocol: initial spins at 2,000 g for 20 min and 10,000 g for 20 min at 4 °C were used to remove cells and larger vesicles.

Finally, exosomes were collected by ultracentrifuging this supernatant at 110,000 g for 70 min at 4 °C using a Beckman ultracentrifuge (Type 90 Ti rotor), and the pellet was washed with PBS before being ultracentrifuged again at 11,000 g for 70 min at 4 °C.

### Exosome OptiPrep density gradient and LAP-TGF-β1 detection

For gradient construction, we formulated solutions of 50%, 40%, and 10% (w/v) iodixanol by diluting OptiPrep (60% (w/v) aqueous iodixanol, Stemcell) with a mixture of 0.85% NaCl and 10 mM Tris-HCl (pH 7.4). The exosome pellet was resuspended in 50% iodixanol (3.8 mL) and layered into an 8 × 13.5 mL centrifuge tube (Beckman). Subsequently, 3 mL of 40% and 2.5 mL of 10% iodixanol solutions were carefully layered atop using a precision needle. This gradient underwent ultracentrifugation at 200,000 g for 2 h at 4 °C. Ten individual iodixanol fractions, subsequently diluted with PBS, were harvested top-down, centrifuged at 110,000 g for 70 min at 4 °C, and then resuspended in PBS. The density of each fraction was quantified gravimetrically. Exosomes from cells underwent morphological characterization by TEM, particularly those isolated from fraction no. 3, as previously cited [27]. Exosome size distribution and concentration were ascertained using the DKSH nanoparticle tracking analyzer (Zetaview, PMX). Expression levels of LAP-TGF-β1 in the exosomes were quantified via the TGF-β1 Quantikine ELISA kit (DB100B, R&D Systems) per manufacturer’s guidelines.

### Mouse experimental procedures

#### Ethical compliance

Animal experiments were executed in strict accordance with protocols sanctioned by the China Pharmaceutical University’s Institutional Animal Care and Use Committee (Permit: 2019-05-004). Post-experiment, mice were euthanized, perfused with PBS, and the requisite tissues were harvested.

#### In vivo exosome distribution

24 h post tail vein injection of PKH67-labeled exosomes (1.25 × 10^10^ particles), organs including the lungs, brains, and livers from female NCG-HLA-A2.1 mice were procured for analyzing exosome uptake via immunofluorescence (IF). To inspect extravasated dextran, these mice, after a 24-hour exosome treatment, received an intravenous dose of 100 mg/kg rhodamine-dextran (MW approx. 70,000), and lung tissues were examined 3 h post-injection.

#### Lung metastasis and orthotopic xenograft models

For the pulmonary metastasis experiment, GFP-Luc-tagged 231 parental/231 LuT3 (5 × 10^5^) cells were intravenously delivered into female NCG-HLA-A2.1 mice aged 6–8 weeks. For the orthotopic mammary xenograft study, 6–8 week-old female NCG-HLA-A2.1 mice received injections of 1 × 10^6^ Luc-marked 231 parental/231 LuT1/231 LuT2/231 LuT3 cells into their No. 4 mammary fat pad. Tumor growth was monitored every third day, using the formula: volume = (length x width^2^)/2. Additionally, BALB/c-nu/nu nude mice of the same age were subcutaneously injected with 5 × 10^6^ A549 cells or LuECs for the subcutaneous xenograft study.

#### Lung metastasis with exosome modulation

Over three weeks, exosomes (1.25 × 10^10^ particles) were intravenously administered every two days to mimic the sustained presence of primary tumors and systematic exosome release. Following this regimen, GFP-Luc-tagged 231 parental/231 LuT3 (5 × 10^5^) cells were introduced intravenously a day after the last exosome dose.

#### Statistical procedures

Statistical analyses were undertaken using GraphPad Prism 8.0. Data representation is as mean ± SD, with details on biological replicates and sample sizes indicated in figure captions. Statistical significance was ascertained using two-tailed Student’s t-test or one-way ANOVA, considering p-values below 0.05 as significant. Software applications ImageJ (1.49p version) and FlowJo (10.8.1 version) were employed for image processing and data analysis, while Photoshop CC (20.0.8 version) and Illustrator CC (26.0.1 version) catered to image refinement and visualization.

## Results

### Exosomes from TNBC Lung metastatic sites remodel the pulmonary vascular niche

**Fig. 1:**
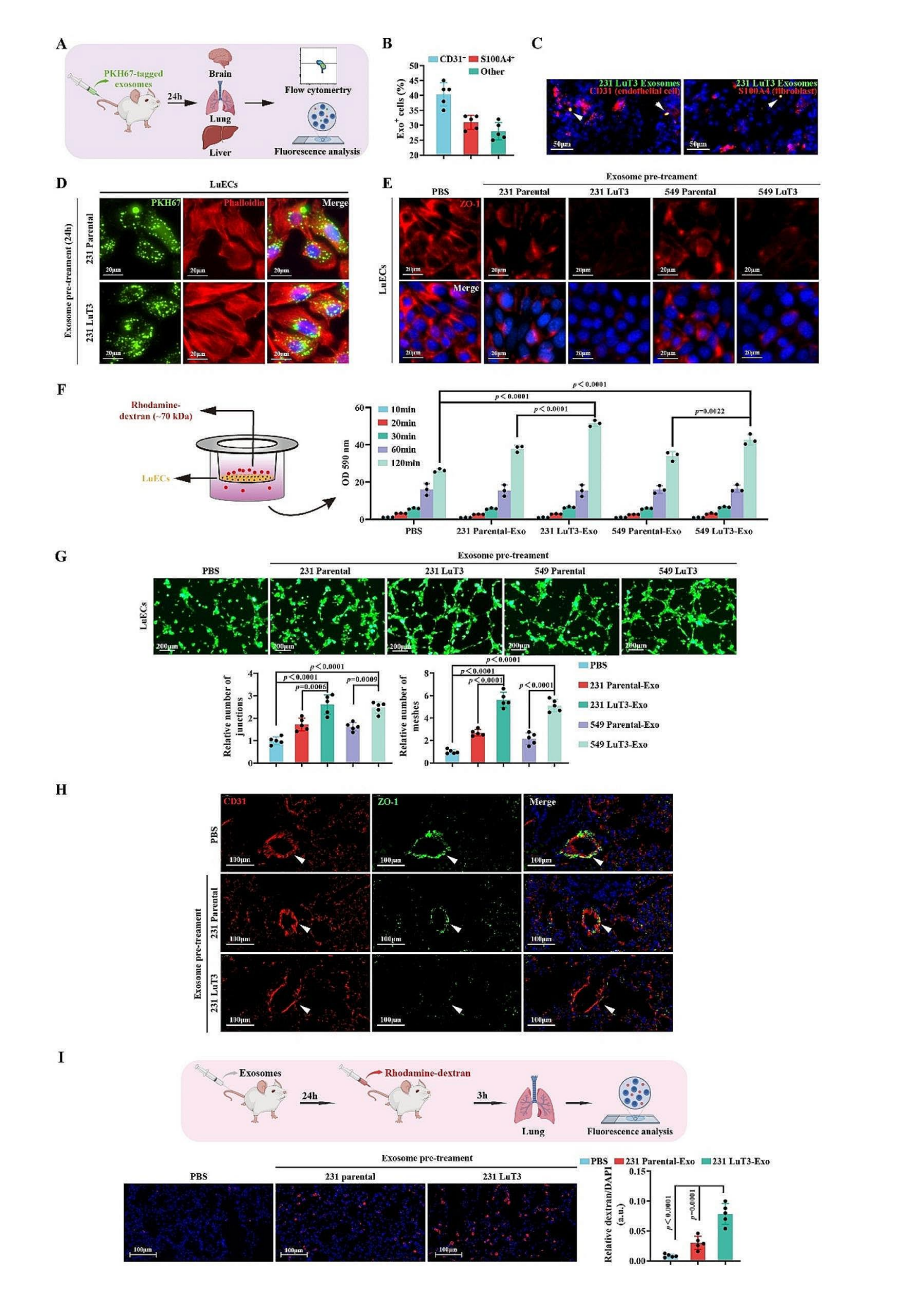
Pulmonary metastatic TNBC-derived exosomes modify the pulmonary vascular niche. (**A**) Schematic representation of organ tissue post-exosome injection in mice. (**B**) Flow cytometry analysis depicting lung resident cell uptake of 231 LuT3 exosomes. (*n* = 5). (**C**) IF co-staining of 231 LuT3 exosomes (green) with either S100A4 (fibroblasts) or CD31 (endothelial cells) in mouse lung samples. Scale bars: 50 μm. (**D**) LuEC treatment with PKH67-tagged exosomes (green) for 24 h, counterstained using Phalloidin (red) and DAPI (blue). Scale bars: 20 μm. (**E**-**G**) LuEC monolayers were treated with either PBS or exosomes for 24 h. (**E**) IF detection of ZO-1 (red). Scale bars: 20 μm. (**F**) Permeability assessment of treated LuEC monolayers on transwell filters using 20 mg/mL rhodamine-dextran (*n* = 3). (**G**) Evaluation of the tube formation (*n* = 5). Scale bars: 200 μm. (**H**) IF double-labeling of CD31 (red) and ZO-1 (green) in mouse lung tissues post 24-hour exosome treatment. Scale bars: 100 μm. (**I**) In vivo visualization of pulmonary vascular permeability via rhodamine-dextran (red) presence (*n* = 5). Scale bars: 100 μm. Data shown as mean ± SD

To understand the influence of exosomes from TNBC lung metastatic sites on the lung microenvironment, we intravenously injected MDA-MB-231 parental (231 Parental) or BT-549 parental (549 Parental) cells into NCG-HLA-A2.1 mice. Following three in vivo selection rounds, we isolated breast cancer subgroups established at lung metastatic sites, designated as 231 LuT and 549 LuT cells, respectively (Fig. S1A). Transwell assays indicated that 231 LuT and 549 LuT cells, post three consecutive in vivo selections, displayed progressively enhanced migratory capacities compared to their 231 Parental and 549 Parental counterparts (Fig. S1B-C). Moreover, nanoparticle tracking analysis (NTA) revealed no significant disparities in the size or quantity of exosomes released by equivalent counts of 231 Parental, 231 LuT3, 549 Parental, and 549 LuT3 cells (Fig. S2A). To discern the specific organ tropism of these breast cancer-derived exosomes, PKH67-labeled exosomes from 231 Parental and 231 LuT3 cells were injected into NCG-HLA-A2.1 mice. Their biodistribution and uptake were then evaluated using flow cytometry and fluorescence analyses (Fig. 1A). Fluorescent tracking showcased a pronounced lung localization for exosomes secreted by 231 LuT3 cells, with their uptake efficiency in the lungs being 2.8-fold that of 231 Parental-derived exosomes (Fig. S2B). Additionally, PKH67-labeled 231 LuT3 exosomes prominently co-localized with lung CD31^+^ endothelial cells and S100A4^+^ fibroblasts, comprising approximately 40% and 30% of exosome-positive cells, respectively (Fig. 1B). Immunofluorescence further corroborated that a larger subset of pulmonary endothelial cells internalized the 231 LuT3-derived exosomes (Fig. 1C). Collectively, these findings insinuate that TNBC lung metastatic exosomes might potentiate TNBC lung micro-metastatic dissemination by architecting the pulmonary vascular niche.

Metastatic tumor progression fundamentally encompasses local tissue invasion, intravasation, circulation in the vascular system, followed by extravasation into distant tissues or organs [28]. Cancer-derived exosomes emerge as prime drivers in instigating pre-metastatic niche attributes, such as angiogenesis and vascular permeability [11, 29, 30]. To probe the influence of lung metastatic exosomes on the pulmonary vascular niche, we isolated lung endothelial cells (LuECs) from immunodeficient mice, as delineated in Fig. S3A. The presence of endothelial cell markers (CD45^−^/CD31^+^ and CD45^−^/VE-cadherin^+^) and the inability to induce subcutaneous xenograft tumors authenticated their identity as LuECs (Fig. S3B-C). Subsequent incubation of LuECs with PKH67-labeled exosomes from either 231 Parental or 231 LuT3 revealed differential uptakes over varying incubation durations, discerned via flow cytometry and immunofluorescence (Fig. S4A-B and Fig. 1D). Since the in vitro study directly applied exosomes to LuECs, the long-term incubation resulted in a high exosome uptake efficiency of LuECs without significant difference. In order to avoid the influence of the difference in the number of ingested exosomes on the function of LuECs, the subsequent experiments were incubated with exosomes for 24 h. Tight junctions (TJs) play a pivotal role in maintaining vascular integrity and dictate endothelial monolayer permeability. The central TJ component, ZO-1, orchestrates copious cell-cell adhesion complexes in endothelial and epithelial cells and has been implicated in breast cancer metastasis [31]. In comparison to PBS and 231 Parental-derived exosomes, 231 LuT3 exosomes suppressed ZO-1 protein expression (Fig. 1E), compromising pulmonary endothelial vascular integrity. This was mirrored by the induced extravasation of rhodamine-labeled dextran probes from apical chambers to basal pores (Fig. 1F), accentuating heightened macromolecular dextran permeability. To determine whether vascular remodeling is instigated by pre-treatment with lung metastatic exosomes, LuECs were exposed to exosomes derived from 231 LuT3, 231 Parental, or PBS. Microscopic imaging underscored that, relative to PBS or 231 Parental exosome treatments, 231 LuT3 exosomes augmented nodal and tubular formations in LuECs (Fig. 1G). To further delineate the role of exosomes from lung metastatic foci in modulating the pulmonary vascular niche, we introduced exosomes derived from either 231 LuT3 or 231 Parental, or PBS (as a control), into NCG-HLA-A2.1 mice and later assessed the mouse lungs via fluorescence analysis. In line with prior findings, compared to PBS and 231 Parental exosomes, 231 LuT3 exosomes significantly suppressed the protein expression of ZO-1 in lung CD31^+^ ECs, impairing pulmonary vascular integrity (Fig. 1H). Concurrently, dextran permeability assays revealed a pronounced enhancement of vascular permeability by the 231 LuT3 exosomes (Fig. 1I).

### Exosomes emitted from TNBC lung metastatic foci propel the dispersal of lung micro-metastatic foci

**Fig. 2:**
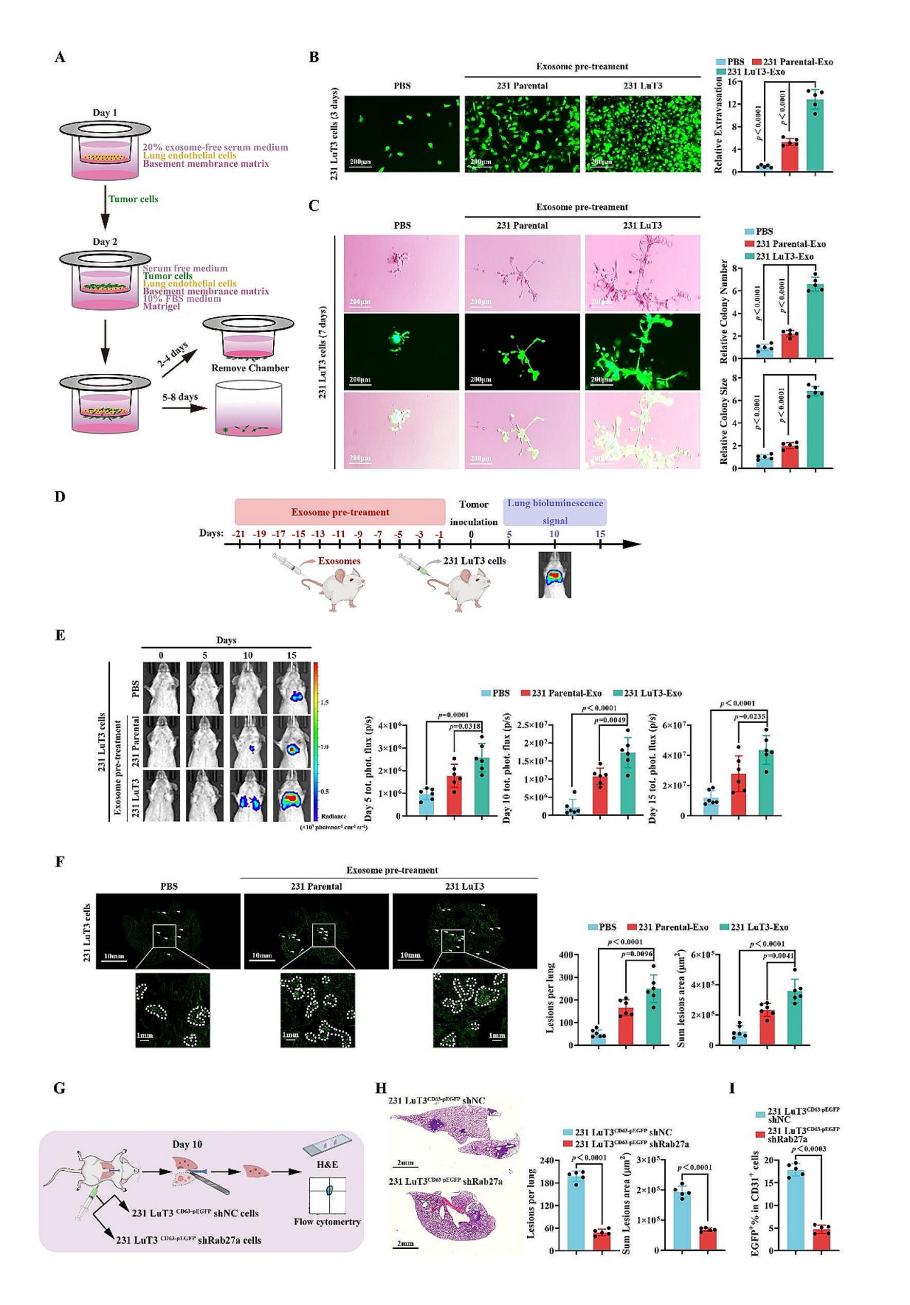
Exosomes from pulmonary metastatic TNBC advance lung micro-metastasis spread. (**A**) In vitro metastasis assay representation simulating extravasation, invasion into distant tissue, and subsequent metastatic colonization/growth of distant locales. (**B**-**C**) After treating LuECs with PBS or 231-derived exosomes for 24 h, GFP-labeled 231 LuT3 cells were implanted into transwell inserts (*n* = 5). Scale bars: 200 μm. (**B**) Extravasation (3 days). (**C**) Invasion through the basement membrane, followed by the development of a mammosphere (7 days). (**D**-**F**) Post 3-week intravenous exosome or PBS injection, mice were further given 5 × 10^5^ GFP-Luc-labeled 231 LuT3 cells intravenously (*n* = 6). (**D**) Schematic of the exosome-induced TNBC lung metastasis model. (**E**) Bioluminescent lung images and metastasis quantification at days 5, 10, and 15 post cell injection (*n* = 6). (**F**) Full lung section images highlighting GFP lung metastasis 15 days post cell administration (*n* = 6). Scale bars: 10 mm and 1 mm. (**G**-**I**) Mouse left lung was inoculated with 231 LuT3^CD63 − pEGFP^ shNC or 231 LuT3^CD63 − pEGFP^ shRab27a cells, and the right lung was harvested after 10 days (*n* = 5). (**G**) Diagram detailing the TNBC lung micro-metastasis dissemination animal model. (**H**) Images of the mouse right lung, with quantitative lung metastatic lesion analysis (*n* = 5). (**I**) Endothelial cell exosome uptake in the right lung via flow cytometry (*n* = 5). Data shown as mean ± SD

Subsequently, we established an in vitro model mimicking TNBC pulmonary metastatic dissemination [32], which simulated TNBC cell extravasation from the lung circulation system (transendothelial migration through LuECs) followed by metastatic colonization/growth (breast spheroid formation) (Fig. 2A). In comparison to 231 Parental cells, 231 LuT3 cells exhibited a similar growth rate (Fig. S5A), yet demonstrated a marked increase in extravasation and subsequent metastatic colonization (Fig. S5B-C), suggesting an augmented proclivity for lung colonization by the lung metastatic foci cells. After pre-treatment of LuECs with exosomes from 231 LuT3 and 231 Parental, or 549 LuT3 and 549 Parental for 24 h, the transendothelial migration and colonization of 231 LuT3 or 549 LuT3 cells were assessed on days 3 and 7. Contrasted with PBS and parental cell-derived exosomes, exosomes from lung metastatic foci notably augmented both the extravasation and colonization capabilities of lung metastatic foci cells (Fig. 2B-C and Fig. S6A-B). These observations underscore that pre-treatment of LuECs with exosomes from lung metastatic foci cells potentiates tumor colonization independent of the intrinsic metastatic potential of cells, suggesting that exosome-mediated pulmonary vascular niche remodeling bolsters re-dissemination of metastatic foci cells.

While the aforementioned studies suggest that exosomes from lung metastatic foci promote extravasation and colonization, the data were primarily grounded in partial in vitro emulation of the metastatic cascade. To delve deeper into this in an in vivo context, we utilized mice acclimatized to exosomes from lung metastatic foci. Prior to the injection of 231 LuT3^GFP − Luc^ cells, the mice were administered either 231 LuT3 or 231 Parental-derived exosomes every other day for three weeks (Fig. 2D). Bioluminescence imaging (BLI) revealed that, in comparison to PBS or 231 Parental exosome pre-treatment, the 231 LuT3 exosome regimen markedly accelerated pulmonary metastasis on days 5, 10, and 15 post-injection of 231 LuT3^GFP − Luc^ cells (Fig. 2E). Quantification of pulmonary lesions confirmed a substantial uptick in the number and area of pulmonary metastatic foci in mice treated with 231 LuT3 exosomes relative to PBS and 231 Parental exosome-treated cohorts (Fig. 2F). Rab27a significantly influences the fusion between multivesicular bodies and the plasma membrane, thereby directly affecting the efficiency and velocity of exosome secretion [33]. To further gauge the exosomal influence on pulmonary micro-metastatic tumor dispersion, 231 LuT3^CD63 − pEGFP^ shNC or 231 LuT3^CD63 − pEGFP^ shRab27a cells were grafted onto the left lung lobes of mice, and ten days later, the right lung lobes were subjected to H&E and flow cytometry analyses (Fig. 2G). Both 231 Parental^CD63−pEGFP^ and its lung metastatic offspring, 231 LuT^CD63−pEGFP^ cells, secreted EGFP^+^ exosomes. H&E staining and flow cytometry data substantiated that following Rab27a knockdown, there was a considerable decline in the number and area of metastatic foci in the right lung lobes of mice (Fig. 2H). The fusion ratio of endothelial cells (CD31^+^) to exosomes (EGFP^+^) was also diminished (Fig. 2I). Collectively, these findings bolster the notion that exosomes from lung metastatic foci facilitate the in vivo dispersal of TNBC pulmonary micro-metastatic foci.

### LAP-TGF-β1 as a functional exosomal content promoting the dispersal of TNBC pulmonary micro-metastatic foci

**Fig. 3:**
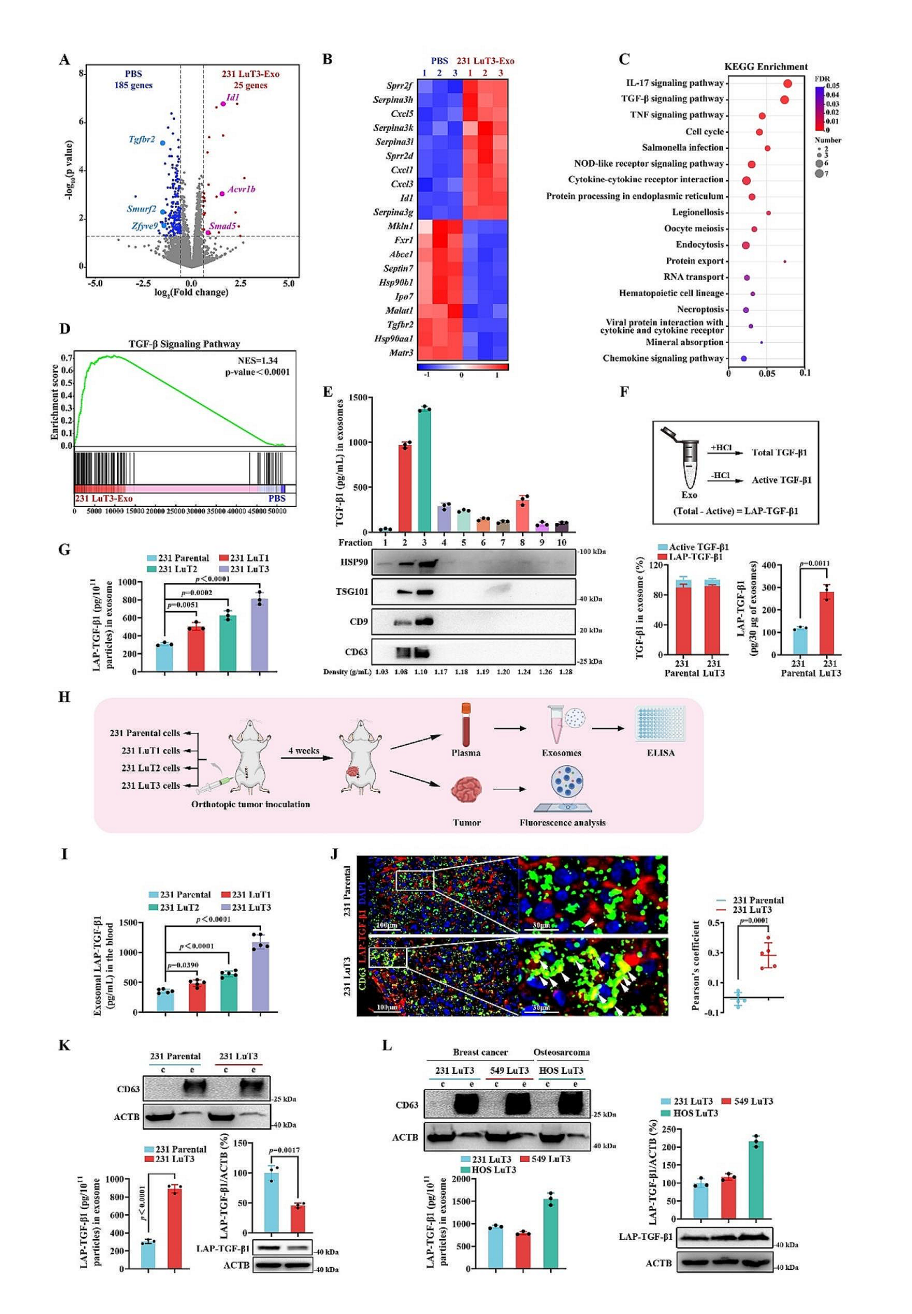
High LAP-TGF-β1 protein levels in exosomes from pulmonary metastatic cells. (**A**) Volcano plot showcasing transcriptome shifts in LuECs after treatment with 231 LuT3 exosomes. Significant genes (*p* < 0.05, fold change > 1.5) highlighted. (**B**) Heatmap representation of gene expression shifts in LuECs post 24-hour exosome treatment. (*n* = 3). (**C**) KEGG pathway analysis for significantly altered genes upon exosome treatment. (**D**) GSEA for TGF-β pathway-enriched genes post exosome treatment. (**E**) Expression analysis of TGF-β1 and exosomal markers in a density gradient of 231 LuT3 exosomes in iodixanol (*n* = 3). (**F**) Comparison of active and inactive TGF-β1 forms in 231-derived exosomes (*n* = 3). (**G**) LAP-TGF-β1 concentration in exosome pellets. (*n* = 3). (**H**-**J**) GFP-Luc labeled 231-derived cells were injected into NCG-HLA-A2.1 mice, with tumors and plasma harvested 17 days later (*n* = 5). (**H**) Breast cancer orthotopic model schematic. (**I**) LAP-TGF-β1 levels in NCG-HLA-A2.1 mouse plasma exosomes (*n* = 5). (**J**) IF imaging of LAP-TGF-β1 and CD63 (*n* = 5). Scale bars: 100 μm and 30 μm. (**K**-**L**) Analysis of LAP-TGF-β1 and CD63 levels in cellular and exosomal proteins (*n* = 3). Data shown as mean ± SD

To dissect the molecular alterations induced by lung metastatic foci exosomes during pulmonary vascular niche remodeling, we employed RNA sequencing to probe the gene expression landscape of LuECs exposed to either PBS or 231 LuT3 exosomes. Contrasted with the PBS group, pretreatment with 231 LuT3 exosomes elicited transcriptional shifts in numerous transcripts within LuECs, notably with elevated transcript levels of Id1 and a downregulated Tgfbr2 (Fig. 3A-B). Moreover, Kyoto Encyclopedia of Genes and Genomes (KEGG) pathway analysis revealed a significant enrichment of genes linked to the TGF-β-associated pathway in LuECs treated with 231 LuT3 exosomes (Fig. 3C). This assertion was further corroborated through gene set enrichment analysis (GSEA) targeting key genes governing TGF-β signaling (Fig. 3D).

TGF-β serves as a pivotal signaling conduit within the tumor microenvironment, with pathway activity principally steered by TGF-β1 activation present within tumors [34]. To pinpoint the TGF-β1 protein within 231 LuT3 exosomes, a conjunction of ultra-high-speed centrifugation with iodixanol/Optiprep density gradient centrifugation was employed. Both ELISA and western blot analyses revealed the presence of TGF-β1 in exosomal fractions marked positive for exosomal markers HSP90, TSG101, CD63, and CD9, with fraction 3 demonstrating the highest expression level (density approximately 1.10 g/mL) (Fig. 3E). TGF-β1 manifests in both active (active TGF-β1) and latent (LAP-TGF-β1) forms, distinguishable through hydrochloric acid (HCl) acidification followed by TGF-β1 ELISA (Fig. 3F). Contrary to the typical exosomal TGF-β1 composition from normal cells (mast cells) – approximately 60% latent and 40% active [24]– both 231 Parental and 231 LuT3 exosomes predominantly harbor TGF-β1 in its latent form (∼ 90%) (Fig. 3F). Around 120 pg and 290 pg of LAP-TGF-β1 were discerned in 30 µg of 231 Parental and 231 LuT3 exosomes, respectively, amounting to 11 and 27 times that of adipocyte-derived exosomes (Fig. 3F) [24]. Further quantitation of LAP-TGF-β1 in exosomes from lung metastatic foci obtained post three in vivo rounds of selection illustrated a sequential escalation in LAP-TGF-β1 content across 231 LuT1, 231 LuT2, and 231 LuT3 exosomes (Fig. 3G). Following this, 231 Parental, 231 LuT1, 231 LuT2, and 231 LuT3 cells were inoculated into the fourth mammary fat pad of NCG-HLA-A2.1 mice, with exosomal LAP-TGF-β1 levels and tumor tissue assessed post four weeks (Fig. 3H). ELISA data showcased notably elevated plasma exosomal LAP-TGF-β1 levels in the 231 LuT2 and 231 LuT3 groups compared to the 231 Parental cohort (Fig. 3I). Double-label IF staining of 231 LuT3 tumor tissue unveiled a significant uptick in colocalization of CD63 and LAP-TGF-β1 as opposed to 231 Parental tumor tissue (Fig. 3J). More intriguingly, the LAP-TGF-β1 content in 231 LuT3 exosomes considerably overshadowed that in 231 Parental exosomes, despite the inverse being true for cellular expression levels (Fig. 3K). This insinuates that the enhanced exosomal loading of LAP-TGF-β1 stems from an amplified active transport mechanism rather than cellular overexpression. Moreover, exosomes derived from 549 LuT3 cells and osteosarcoma lung metastatic foci (HOS LuT3) cells displayed pronounced LAP-TGF-β1 expression (Fig. 3L), further corroborating the intimate association between elevated exosomal LAP-TGF-β1 loads in lung metastatic foci and tumor micro-metastatic foci dissemination within the lung.

### Exosomal LAP-TGF-β1 drives pulmonary vascular niche remodeling and propels dispersal of pulmonary micrometastatic foci

To delineate whether the exosome-mediated dispersal of pulmonary micrometastatic foci is contingent on LAP-TGF-β1, we harnessed CRISPR-Cas9 to target and abrogate TGF-β1 in lung metastatic foci 231 LuT3 cells. Validated by Western blot and ELISA, the expression of LAP-TGF-β1 was significantly attenuated in two single-cell clones of 231 LuT3 with TGF-β1 knockout (KO), in comparison to the wild-type (WT) 231 LuT3 cells and their exosomes (Fig. S7A). Transmission electron microscopy (TEM) and NTA affirmed that the TGFB1 KO neither altered the morphology, number, nor size of the exosomes (Fig. S7B-C). Additionally, protein levels of CD9, CD63, TSG101, and ALIX remained invariant in the 231 LuT3 TGFB1 KO exosomes, suggesting that the loss of TGFB1 does not modify exosomal protein packaging (Fig. S7A).

**Fig. 4:**
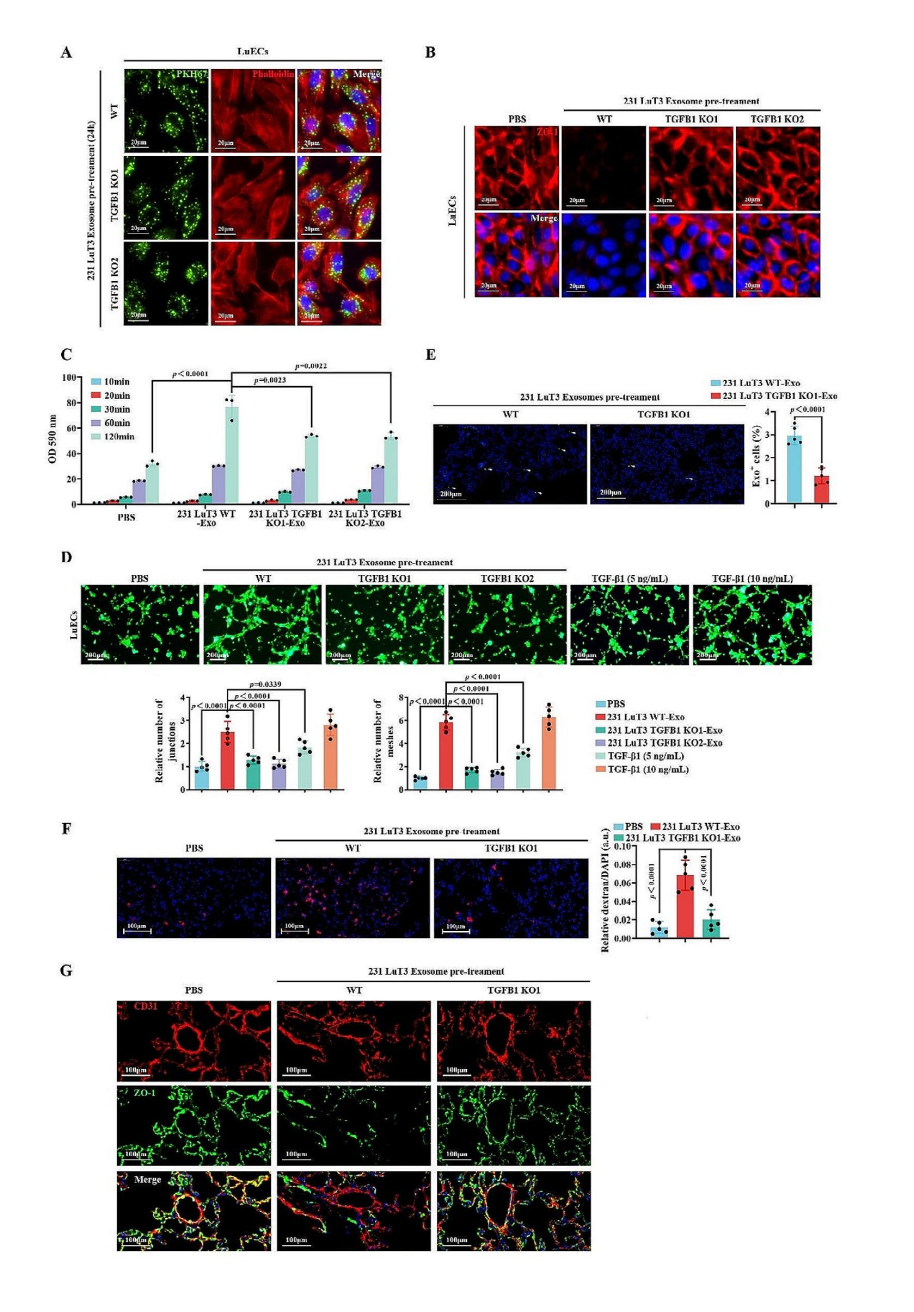
Exosomal LAP-TGF-β1 reshapes the pulmonary vascular environment. (**A**) After 24 h of treatment with PKH67-labeled exosomes, LuECs were stained with Phalloidin and DAPI. Scale bars: 20 μm. (**B**-**D**) 24-hour treatment of LuEC monolayers with PBS or exosomes or TGF-β1. (**B**) IF detection of ZO-1. Scale bars: 20 μm. (**C**) Permeability assessment using 20 mg/mL rhodamine-dextran on treated LuEC monolayers on transwell filters (*n* = 3). (**D**) Tube formation assay (*n* = 5). Scale bars: 200 μm. (**E**) Biodistribution of PKH67-labeled exosomes in mouse lungs (*n* = 5). Arrows indicate exosome. Scale bars: 200 μm. (**F**) In vivo vascular permeability visualized via rhodamine-dextran (*n* = 5). Scale bars: 100 μm. (**G**) Double-label IF of CD31 and ZO-1 in mouse lungs post-exosome treatment. Scale bars: 100 μm. Data shown as mean ± SD

Subsequently, we explored the function of exosomal LAP-TGF-β1 within the pulmonary vascular niche. Although a 6-hour incubation with PKH67-labeled exosomes revealed that TGFB1 KO decreased the uptake of 231 LuT3 exosomes by LuECs (Fig. S8A-B), it had no influence on exosomal uptake observed after a 24-hour incubation (Fig. 4A and S8B). After 24 h of treating LuECs with exosomes, it was evident that the TGFB1 KO significantly mitigated the inhibitory effect of TNBC pulmonary metastatic foci exosomes on ZO-1 protein expression (Fig. 4B) and hindered the exosome-induced extravasation of high-molecular-weight dextran (Fig. 4C). Furthermore, through an angiogenesis assay, we ascertained the role of exosomal LAP-TGF-β1 in vascular remodeling. Relative to 231 LuT3 TGFB1 WT exosomes, the nodal and tubular structures formed by LuECs treated with 231 LuT3 TGFB1 KO exosomes were reduced, and 10 ng/mL of free TGF-β1 exhibited effects parallel to the pulmonary metastatic foci exosomal TGF-β1 (Fig. 4D). To gain deeper insights into the role of exosomal LAP-TGF-β1 within the pulmonary vascular niche, mice were intravenously administered with 231 LuT3 TGFB1 WT or 231 LuT3 TGFB1 KO1 exosomes. In comparison to the 231 LuT3 TGFB1 WT group, the absence of TGFB1 in 231 LuT3 exosomes led to a 2.4-fold reduction in pulmonary uptake efficiency (Fig. 4E). Additionally, the loss of TGFB1 suppressed the deteriorative effect of pulmonary metastatic foci exosomes on lung vascular integrity, as evidenced by reduced extravasation of high-molecular-weight dextran and augmented ZO-1 protein expression in pulmonary CD31^+^ ECs (Fig. 4F-G).

**Fig. 5:**
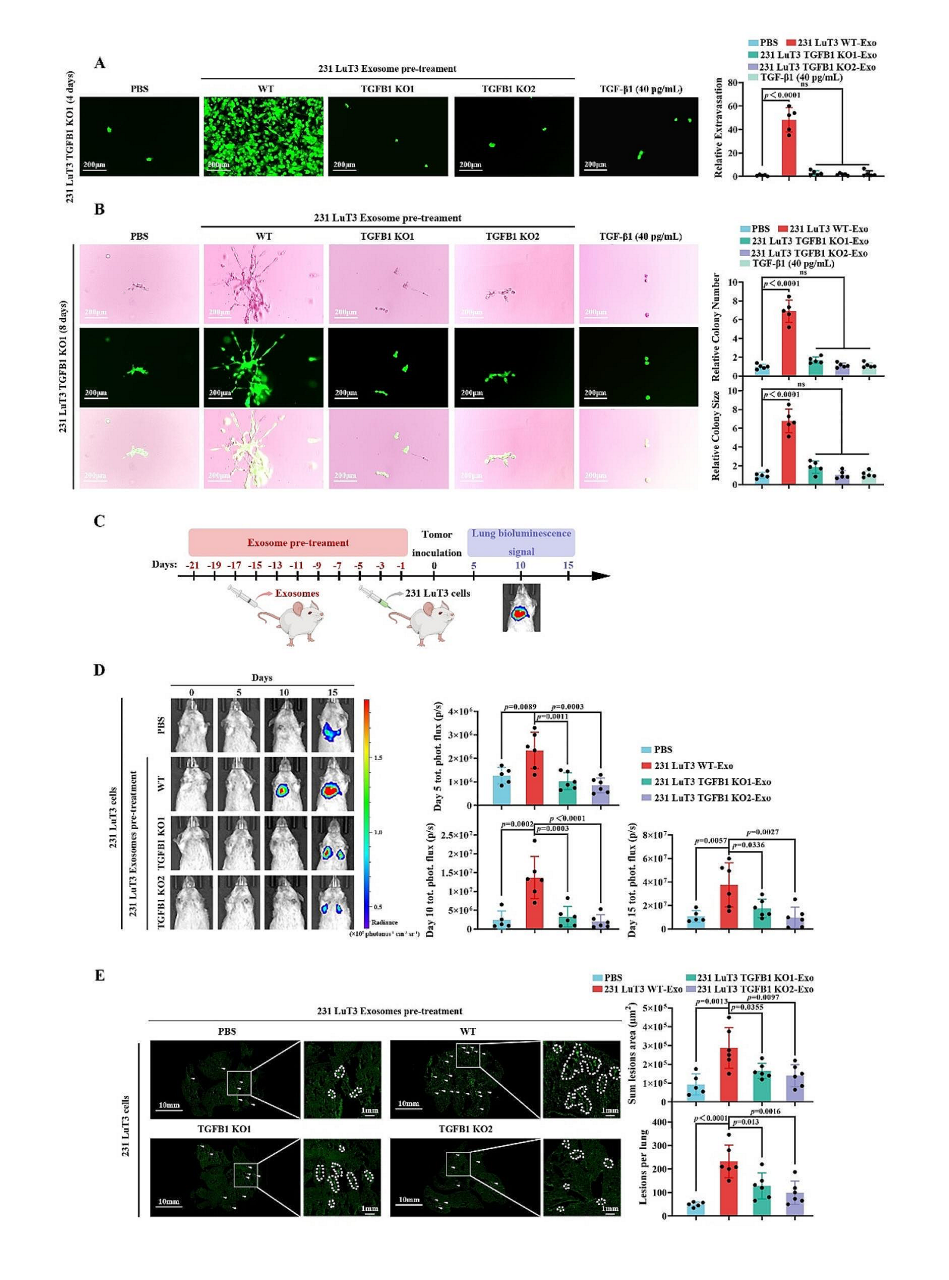
Role of exosomal LAP-TGF-β1 in lung micro-metastases propagation. (**A**-**B**) After treating LuECs with PBS or exosomes or TGF-β1 for 24 h, GFP-labeled 231 LuT3 TGFB1 KO1 cells were implanted into transwell inserts (*n* = 5). Scale bars: 200 μm. (**A**) Exhibits extravasation on day 4; (**B**) Shows the 8-day process of basement membrane invasion and subsequent mammosphere genesis. (**C**-**E**) Post a 3-week intravenous administration of exosomes from either 231 LuT3 or 231 LuT3 TGFB1 KO cells, mice received an intravenous injection of 5 × 10^5^ GFP-Luc-labeled 231 LuT3 cells (*n* = 5–6). (**C**) Schematic representation of the TNBC lung metastasis model influenced by exosomes. (**D**) Depicts luminescent lung images alongside metastases quantification on days 5, 10, and 15 post cell injection. (**E**) By day 15 post-injection, a visual representation of full lung sections reveals GFP-tagged metastases; quantification of total metastatic area and lesion count per lung is presented. Scale bars: 10 mm and 1 mm. Data shown as mean ± SD

Building on prior results highlighting that the remodeling of the pulmonary vascular niche underpins the dispersal of metastatic cells, we scrutinized the role of LAP-TGF-β1 in the dissemination of pulmonary micrometastases. Relative to the 231 LuT3 WT cells, the 231 LuT3 TGFB1 KO cells exhibited markedly diminished extravasation and metastatic colonization (Fig. S9A-B), without any alteration in their proliferation (Fig. S9C-D). These observations suggest that the role of LAP-TGF-β1 in the dissemination within the pulmonary niche is intricately linked to the vascular remodeling. To discern whether exosomal LAP-TGF-β1 was integral to this process, LuECs were pre-treated with 231 LuT3 TGFB1 KO exosomes. As depicted in Fig. S10A-B, the TGFB1 KO considerably dampened the extravasation and colonization triggered by exosomes derived from 231 LuT3. Additionally, to ascertain the relative contributions of exosomal LAP-TGF-β1 and cell-secreted TGF-β1 to pulmonary micro-metastatic dispersal, we investigated whether free TGF-β1 (at a dose equating to 40pg/mL in exosomes) could rescue the extravasation and colonization capabilities of 231 LuT3 TGFB1 KO cells. Microscopic imaging revealed that LuECs treated with 231 LuT3 TGFB1 WT exosomes exhibited pronounced extravasation and colonization by the 231 LuT3 TGFB1 KO1 cells, a phenomenon not observed when LuECs were treated with 231 LuT3 TGFB1 KO exosomes or free TGF-β1 (Fig. 5A-B). This highlights the superiority of exosomal TGF-β1 at lower doses in remodeling the pulmonary vascular niche, ultimately facilitating extravasation and colonization.

To delve deeper into the in vivo role of LAP-TGF-β1 in pulmonary micro-metastatic dispersal, we intravenously injected mice with either 231 LuT3 TGFB1 WT or 231 LuT3 TGFB1 KO cells (Fig. S11A). Pulmonary BLI signal enrichment revealed a significant reduction in lung metastasis 20 days post-injection with 231 LuT3 cells when LAP-TGF-β1 was deficient (Fig. S11B). Comprehensive lung imaging demonstrated fewer pulmonary metastatic foci and reduced metastatic burden in 231 LuT3 TGFB1 KO cells as opposed to the WT counterparts (Fig. S11C). However, no discernible proliferative difference was observed between TGFB1 WT and TGFB1 KO cells (Fig. S9C), indicating the pivotal role of LAP-TGF-β1 during the early stages of metastatic colonization. To ascertain the in vivo influence of exosomal LAP-TGF-β1 on pulmonary micro-metastatic dispersal, mice were preconditioned every other day with 1.25 × 10^10^ particles of exosomes derived from either TGFB1 WT or TGFB1 KO for three weeks, followed by intravenous injection of 231 LuT3 cells (Fig. 5C). On days 5, 10, and 15 post-injection, a pronounced escalation in lung metastasis was observed in mice preconditioned with TGFB1 WT-derived exosomes compared to those treated with TGFB1 KO-derived exosomes (Fig. 5D). Quantification of lung lesions revealed an increased number and area of metastatic foci in mice pre-treated with TGFB1 WT exosomes compared to the PBS and TGFB1 KO exosome-preconditioned groups (Fig. 5E). Collectively, these data underscore the pro-metastatic role of exosomal LAP-TGF-β1 during the early stages of in vivo colonization, suggesting that exosomal LAP-TGF-β1 may emerge as a therapeutic target for combating TNBC pulmonary micrometastatic dissemination.

### LAP-TGF-β1 is channeled into exosomes through interaction with HSP90A

**Fig. 6:**
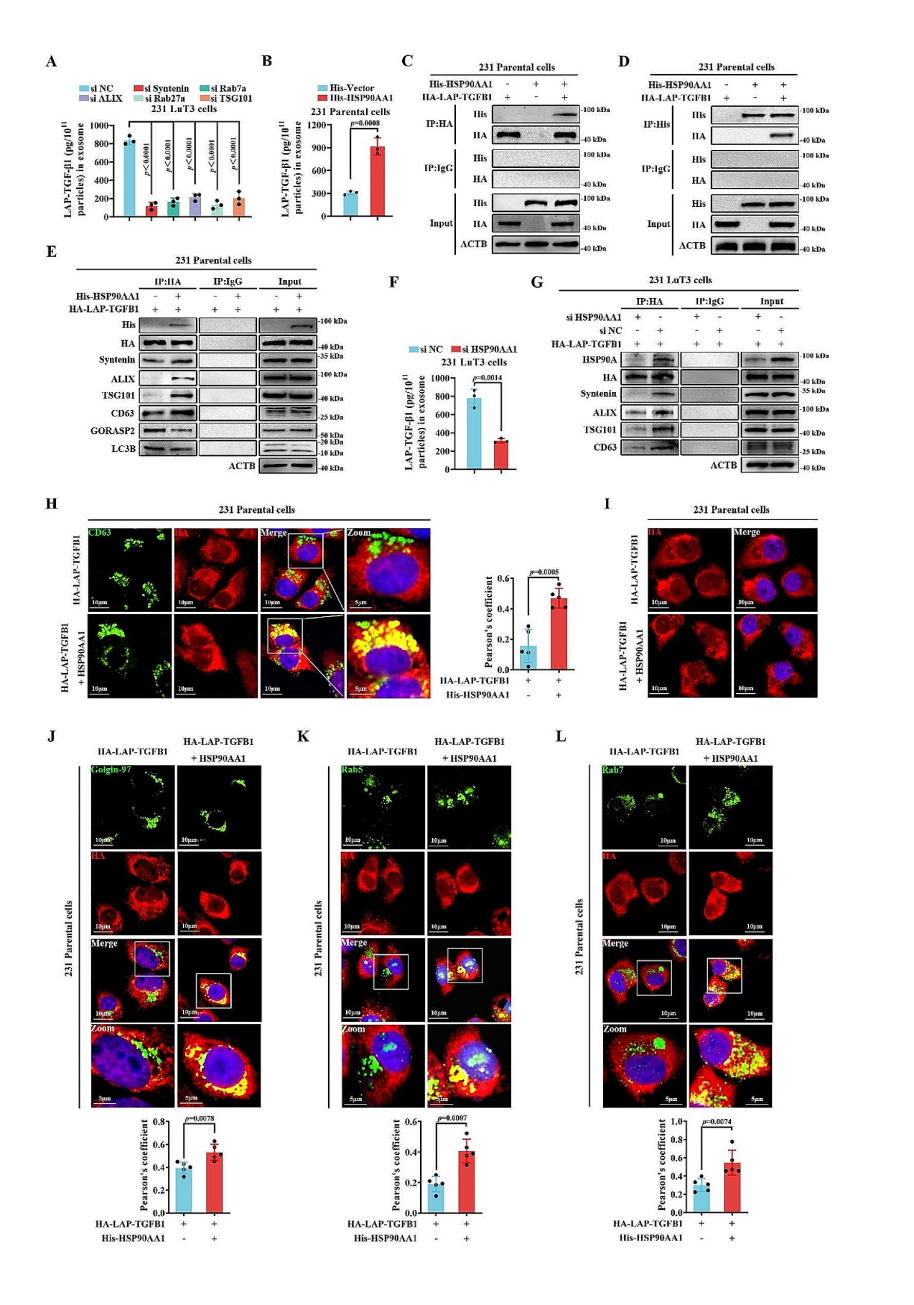
LAP-TGF-β1-HSP90A interaction directs transport from Golgi to endosomes. (**A**) Post-transfection LAP-TGF-β1 exosomal expression from 231 LuT3 cells transfected with designated siRNA (*n* = 3). (**B**) Exosomal LAP-TGF-β1 levels after 231 parental cell transfection with either His-vector or His-HSP90AA1 (*n* = 3). (**C**-**E**) Co-transfection of 231 parental cells with His-HSP90AA1 and HA-LAP-TGFB1, followed by cell purification using specific agarose beads and subsequent antibody-based analysis. (**F**) LAP-TGF-β1-containing exosomes after 231 LuT3 cell transfection with si HSP90AA1 or si NC (*n* = 3). (**G**) HA-LAP-TGFB1 and si HSP90AA1 or si NC were co-transfected into 231 LuT3 cells and purified using anti-HA agarose beads. Immunoprecipitated and immunoblotted cell lysates using the appropriate antibodies. (**H**-**L**) Specific co-transfections and their subsequent intracellular interactions visualized using color-coded labels (*n* = 5). Scale bars: 10 μm and 5 μm. Data shown as mean ± SD

The current repertoire of strategies to inhibit TGF-β signaling pathways, encompassing small molecule inhibitors of TGF-β receptor kinases, TGF-β antibodies, ligand traps, and TGF-β activation inhibitors, has progressed to clinical testing. However, their expansive side effect profiles have hampered clinical applicability [34, 35]. Given the distinctively high enrichment of LAP-TGF-β1 in pulmonary metastatic lesion exosomes, elucidating its loading mechanism and potential intervention might serve as an innovative tactic to tackle the dissemination of pulmonary micrometastases. Utilizing 231 LuT3 cells with high exosomal LAP-TGF-β1 expression, we individually knocked down genes such as SDCBP (Syntenin-1), PDCD6IP (ALIX), Rab7a, Rab27a, and TSG101 using siRNA, all of which are implicated in the biogenesis, transportation, and release of exosomes (Fig. S12) [36–38]. As presented in Fig. 6A, the depletion of these genes resulted in attenuated LAP-TGF-β1 protein expression within 231 LuT3 exosomes, suggesting that LAP-TGF-β1 entry into exosomes leverages an endosomal sorting complex required for transport (ESCRT)-dependent pathway. Given the overexpression of HSP90 in malignancies such as breast cancer and its documented role in facilitating the inclusion of molecules like IL-1β and RAB22A-NEOF1 into vesicles [39, 40], it is plausible that LAP-TGF-β1 might be incorporated into exosomes through an interaction with HSP90A. To determine whether HSP90A was requisite for the secretion of LAP-TGF-β1 into exosomes, 231 Parental cells were transfected with His-HSP90AA1 (Fig. S13A). Despite the number of exosomal particles remaining unchanged (Fig. S13B), there was a significant upregulation of LAP-TGF-β1 within the exosomes (Fig. 6B). Immunoprecipitation assays corroborated the interaction between HSP90A and LAP-TGF-β1 (Fig. 6C-D). While previous studies have denoted the interaction of LAP-TGF-β1 with the Golgi apparatus protein GORASP2, channeling it for transportation into autophagosome intermediaries [41], it is essential to note that exosomal cargo loading is closely intertwined with the Golgi apparatus and endosomes. In 231 Parental cells, overexpression of HSP90AA1 reduced the binding of LAP-TGF-β1 to LC3B and GORASP2 but augmented its association with exosomal-related proteins like Syntenin, ALIX, TSG101, and CD63 (Fig. 6E). This suggests that the interaction between HSP90A and LAP-TGF-β1 favors its entry into exosomes while curtailing its incorporation into autophagosomes. When HSP90AA1 was silenced (Fig. S14A), while the number of released exosomes from 231 LuT3 cells remained relatively stable (Fig. S14B), there was a profound dip in exosomal LAP-TGF-β1 expression (Fig. 6F), and its interaction with exosomal-associated proteins was hampered (Fig. 6G). Dual-labeled IF staining further showcased that HSP90A overexpression facilitated the colocalization of LAP-TGF-β1 and CD63 (Fig. 6H), albeit without inducing LAP-TGF-β1 translocation to the plasma membrane (Fig. 6I). These outcomes highlight that the incorporation of LAP-TGF-β1 into exosomes hinges on its interaction with HSP90A.

TGF-β1 is present on the membrane of the exosome [24], and the exosomal membrane cargo shuttles from the Golgi apparatus to the endosome, where it is then sorted into multivesicular bodies (MVBs) during endosome maturation [42, 43]. With the aid of Golgi marker Golgi-97 staining, we observed enhanced LAP-TGF-β1 expression within the Golgi bodies of 231 Parental cells that overexpressed HSP90AA1 (Fig. 6J). Similarly, in 231 Parental cells, HSP90AA1 overexpression promoted colocalization of LAP-TGF-β1 with the early endosome marker, Rab5 (Fig. 6K). Co-transfection of His-HSP90AA1 and HA-LAP-TGFB1 led to augmented colocalization of LAP-TGF-β1 with Rab7, a marker for late endosomes (Fig. 6L), which subsequently fuse with the plasma membrane to release exosomes [36, 44]. Collectively, these results suggest that HSP90A’s interaction with LAP-TGF-β1 influences the trafficking of LAP-TGF-β1 from the Golgi to endosomes, culminating in its targeting to exosomes.

### LAP-TGF-β1’s interaction with histone acetyltransferase TIP60 at the K304 residue facilitates the dissemination of TNBC pulmonary micrometastatic lesions

**Fig. 7:**
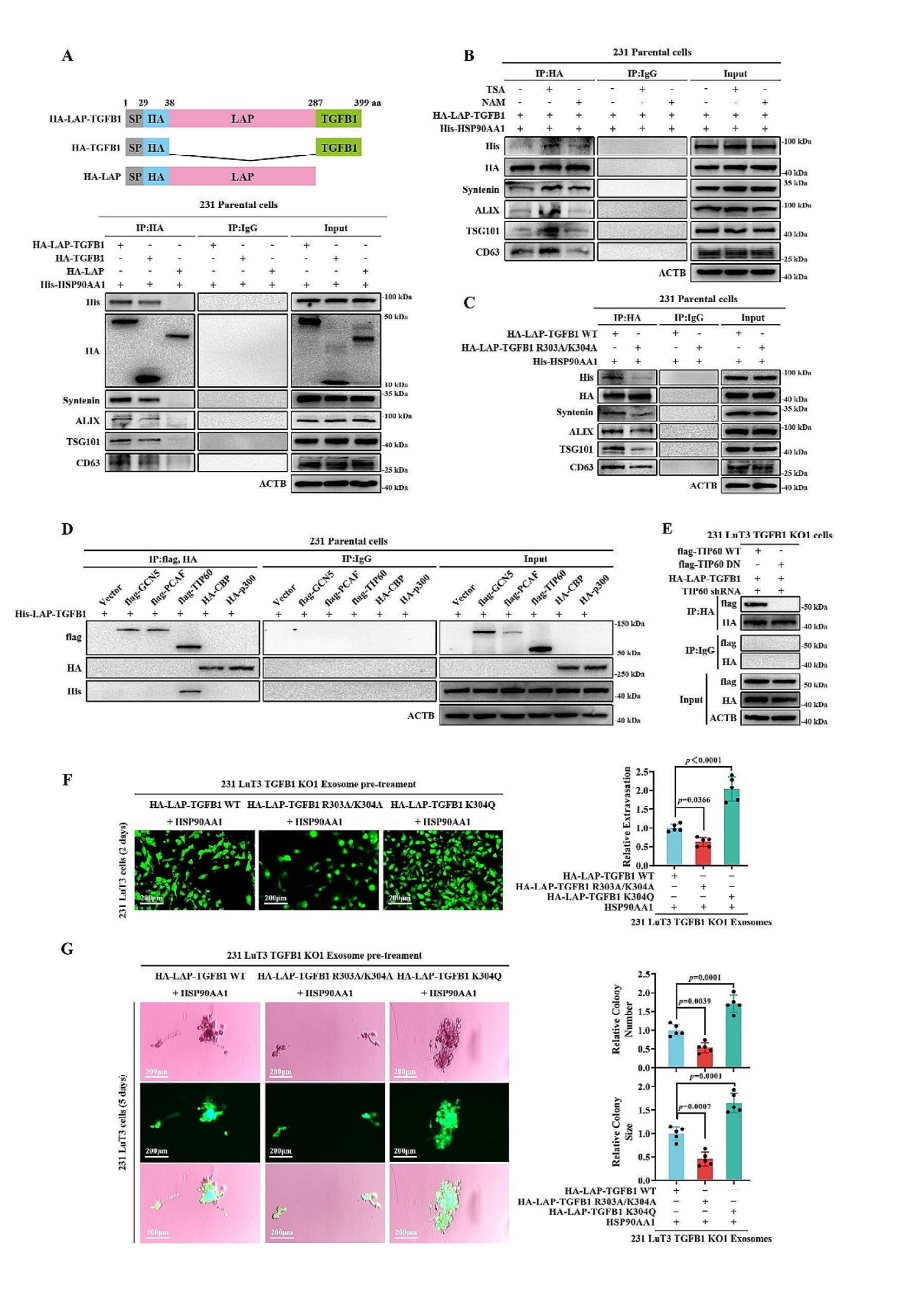
TIP60-driven LAP-TGF-β1 K304 Acetylation Facilitates Lung Metastases Spread. (**A**) Graphic representation of HA-LAP-TGFB1 protein with domain alterations. Following this, 231 parental cells were co-transfected and subsequently processed with designated antibodies. (**B**-**D**) Further explorations of the interactions and modifications of HA-LAP-TGFB1 in response to various treatments. (**E**) The impact of flag-TIP60 variants on HA-LAP-TGFB1 interactions in 231 LuT3 TGFB1 KO1 cells. (**F**-**G**) After treating LuECs with 231 LuT3 TGFB1 KO1-derived exosomes for 24 h, GFP-labeled 231 LuT3 cells were implanted into transwell inserts (*n* = 5). Scale bars: 200 μm. (**F**) Extravasation (2 days). (**G**) Invasion through the basement membrane, followed by the development of a mammosphere (5 days). Data is mean ± SD

In our quest to further validate the interactions between LAP-TGF-β1, HSP90A, and exosomal-associated proteins, and to map the responsible domain within LAP-TGF-β1, we engineered various truncations of LAP-TGF-β1 and scrutinized their co-precipitations with HSP90A and exosomal proteins (Fig. 7A). IP results revealed that the LAP-TGF-β1 lacking the TGF-β1 structural domain displayed diminished interactions with both HSP90A and exosomal-associated proteins (Fig. 7A). Considering the capacity of HSP90A to bolster the secretion of unconventional proteins through its KFERQ-like motif [39, 45], and that the KFERQ sequence generally comprises five residues, our examination of the LAP-TGF-β1 protein sequence pinpointed an atypical acetylated KFERQ-like motif (300IDFRK304) within the TGF-β1 domain. To verify the presence of this acetylation modification, we examined acetylation in ectopically expressed LAP-TGF-β1 in 231 Parental cells treated with broad-spectrum inhibitors of HDAC family deacetylases, Trichostatin A (TSA), and SIRT family deacetylases, nicotinamide (NAM) (Fig. S15A) and found the endogenous LAP-TGF-β1 was acetylated in cells treated with TSA but not NAM (Fig. S15A-B). Furthermore, TSA-treated 231 Parental cells exhibited enhanced binding of LAP-TGF-β1 to both HSP90A and exosomal-associated proteins, in comparison to NAM-treated cells (Fig. 7B). These findings hint at an acetylation modification in LAP-TGF-β1 that potentially drives its entry into exosomes.

Subsequently, we replaced the R303K304 residues with alanine and inspected the mutant’s interaction with HSP90A. In 231 Parental cells, the R303A/K304A mutation significantly curtailed its association with HSP90A and exosomal proteins (Fig. 7C). Additionally, constructing an acetylation-mimicking mutant, HA-LAP-TGFB1 K304Q, we discovered its binding to HSP90A and exosomal proteins was even more potent than HA-LAP-TGF-β1 WT (Fig. S16). Aiming to identify the acetyltransferase responsible for LAP-TGF-β1 acetylation at the K304 site, we examined several prime candidates, including GCN5, PCAF, TIP60, CBP, and p300. Notably, ectopically expressed TIP60 in 231 Parental cells demonstrated a robust specific association with LAP-TGF-β1 (Fig. 7D). Following this line of inquiry, we investigated whether LAP-TGF-β1 acetylation necessitated TIP60. Within 231 LuT3 TGFB1 KO1 cells, an acetyltransferase-deficient TIP60 (TIP60-DN) nullified the interaction between LAP-TGF-β1 and TIP60 (Fig. 7E), suggesting that the acetylation-directed sorting of LAP-TGF-β1 into exosomes is orchestrated by TIP60.

We evaluated the protein expression levels of HSP90A and TIP60 within tumor tissues originating from inoculated 231 Parental and 231 LuT3 cells. Immunoblotting results revealed an upregulation of HSP90A and TIP60 expression in tumor tissues from the 231 LuT3 inoculation group compared to the 231 Parental group (Fig. S17). Exploring the clinical significance of HSP90A and TIP60 in TNBC using the PROGgeneV2 Pan-Cancer Prognostic Database (http://www.progtools.net/gene/), we found that TNBC patients with joint high expression of HSP90A and TIP60 exhibit shorter survival times (Fig. S18). Notably, the LAP-TGFB1 R303A/K304A mutation significantly reduced the exosomal induction of extravasation and colonization of 231 LuT3 cells by exosomes from 231 LuT3 TGFB1 KO1 cells overexpressing LAP-TGFB1 and HSP90AA1 (Fig. 7F-G) and elevated ZO-1 expression in LuECs (Fig. S19). Conversely, the LAP-TGFB1 K304Q mutation augmented these effects (Fig. 7F-G) and reduced ZO-1 expression in LuECs (Fig. S19). Collectively, these results suggest that LAP-TGF-β1 is loaded into exosomes via binding to HSP90A, mediated by the TIP60-regulated acetylation of the KFERQ-like motif (300IDFRK304). This results in the remodeling of pulmonary vascular niches, thereby enhancing the dissemination of pulmonary micrometastatic lesions.

### Dual inhibition of HSP90A and TIP60 effectively impedes TNBC pulmonary micrometastasis dissemination

**Fig. 8:**
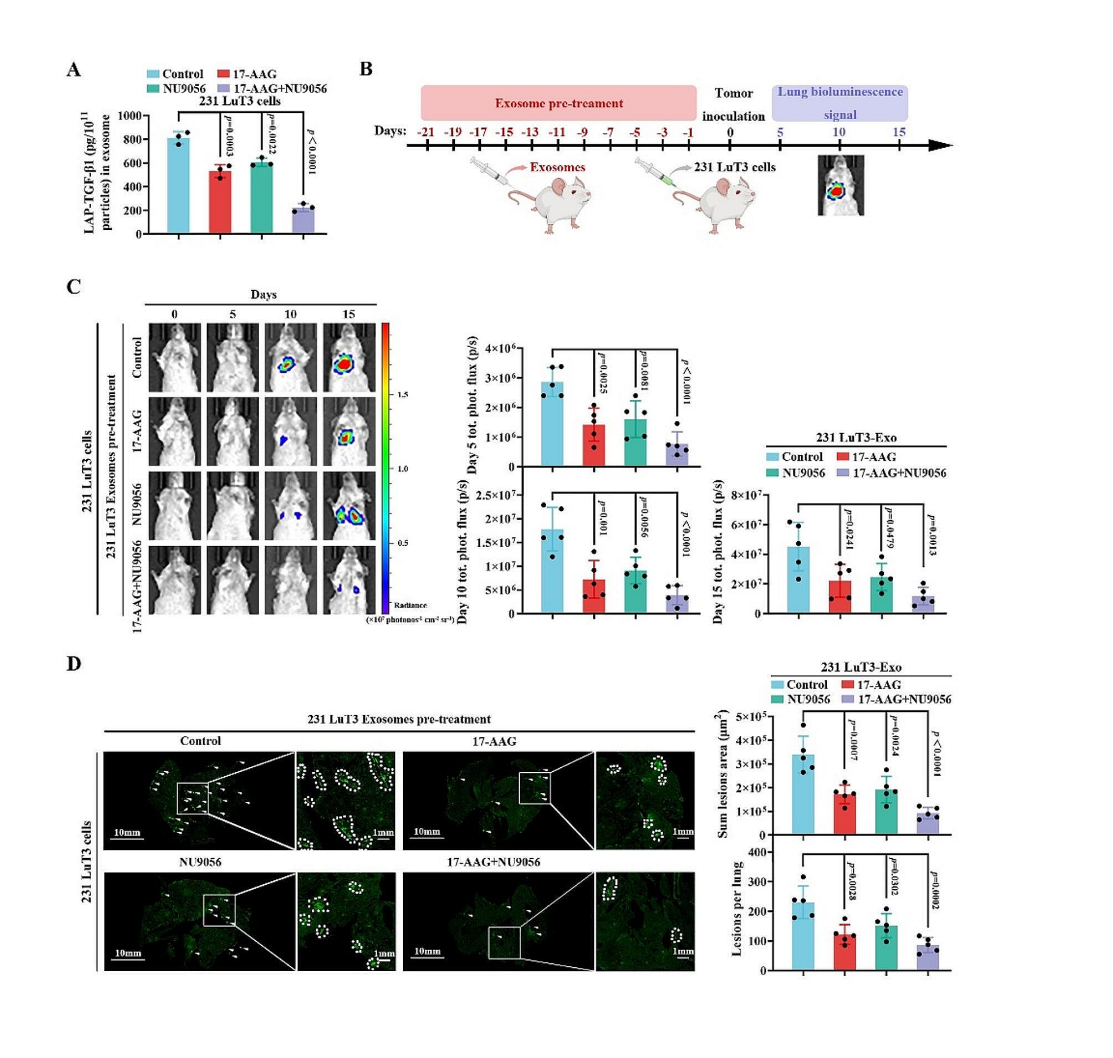
Combined HSP90A and TIP60 inhibition deters TNBC lung micro-metastases. (**A**) Exosomal LAP-TGF-β1 levels post 231 LuT3 cell treatment with specific inhibitors (*n* = 3). (**B**-**D**) After three weeks of intravenous injection of exosomes released from 231 LuT3 cells treated with 17-AAG and/or NU9056, mice were further intravenously injected with 5 × 10^5^ GFP-Luc-labeled 231 LuT3 cells (*n* = 5). (**B**) Schematic representation of the TNBC lung metastasis model influenced by exosomes. (**C**) Depicts luminescent lung images alongside metastases quantification on days 5, 10, and 15 post cell injection. (**D**) By day 15 post-injection, GFP lung metastasis visual documentation and subsequent lesion quantifications. Scale bars: 10 mm and 1 mm. Data is mean ± SD

Previous studies have identified synergistic anti-tumor effects achieved by the combined induction of apoptosis using HSP90 and acetyltransferase inhibitors [46], though their non-cytotoxic actions remain unveiled. Given that HSP90A and TIP60 drive LAP-TGF-β1 into exosomes, we harvested exosomes from 231 LuT3 cells treated with inhibitors targeting HSP90A and/or TIP60 (17-AAG and NU9056). ELISA data indicated that both 17-AAG and NU9056 led to decreased LAP-TGF-β1 protein levels in the exosomes (Fig. 8A). Additionally, at these dosages, 17-AAG and NU9056 exhibited no significant effect on the vitality of 231 LuT3 cells (Fig. S20A) and did not induce apoptosis (Fig. S20B). To investigate the combined effects of HSP90A and TIP60 inhibitors on TNBC pulmonary micrometastasis dissemination, we intravenously injected mice with exosomes derived from 231 LuT3 cells treated with 17-AAG and/or NU9056, bi-weekly for three weeks, followed by an inoculation of 231 LuT3 cells (Fig. 8B). Enhanced BLI signals within the lungs of the mice showed a substantial suppression of pulmonary metastasis promoted by exosomes on days 5, 10, and 15 post-injection of 231 LuT3GFP-Luc cells when treated with the combination of 17-AAG and NU9056 compared to the PBS group (Fig. 8C). Whole-lung imaging demonstrated that this combination markedly reduced both the number and size of pulmonary metastatic lesions, augmented by exosomes, compared to the PBS group (Fig. 8D). Overall, our findings offer the first report that dual inhibition of HSP90A and TIP60, under non-cytotoxic conditions, considerably reduces LAP-TGF-β1 exosomal payload, effectively curtailing the dissemination of pulmonary micrometastatic lesions.

## Discussion

A deeper understanding of the mechanisms underpinning the dissemination of pulmonary micrometastatic lesions, especially the contribution of tumor-derived exosomes, paves the way for targeted interventions for lung metastasis. Our study reveals that preconditioning the pulmonary microenvironment with exosomes from lung metastatic foci cells establishes vascular niches conducive to metastatic colonization. The restructuring of pre-metastatic pulmonary vascular niches is vital in the extravasation-survival phase of pulmonary micrometastatic cells [47]. Vascular remodeling, both in micrometastasis and macrometastasis, could serve as a mechanism of tumor vascularization, a trait tapped into by various cancers—including breast, intestinal, and renal—upon lung metastasis. This hints at the potential role of the pulmonary microenvironment in driving this cancer cell behavior [48, 49]. As circulating cancer cells must traverse the vascular endothelium to reach distant organs [50] and given that the permeability of endothelial cells largely relies on tight junctions (TJs) [51, 52], TJs represent the first barrier cancer cells must navigate during metastasis [53]. We identified LAP-TGF-β1, a protein expressed in the lungs and implicated in conditions such as pulmonary arterial hypertension and acute lung injury [23, 54, 55], as being enriched in exosomes from pulmonary metastatic foci. Despite the acknowledged role of cell-expressed LAP-TGF-β1 in a myriad of physiological, pathological processes, and disease etiologies [56–61], our research underscores that exosomal LAP-TGF-β1 impairs TJ-related protein ZO-1 expression in pulmonary endothelial cells, compromising pulmonary vascular integrity. This fosters vascular remodeling, thus aiding the dissemination of pulmonary micrometastatic foci. The absence of LAP-TGF-β1 curtails the disruptive effects of exosomes from pulmonary metastatic foci on the pulmonary vascular niche and reduces the number of lung metastatic colonies induced by these exosomes in experimental lung metastasis settings. These findings indicate that LAP-TGF-β1-rich exosomes drive metastatic colonization by remodeling the pulmonary vascular niche.

Interestingly, the vasculature-remodeling effect of a high dosage (10 ng/mL) of free TGF-β1 was on par with that of a low dose (40 pg/mL) of exosomal TGF-β1. Only the exosomal form of TGF-β1, not its free counterpart at equivalent dosages, was competent in reinstating the extravasation and pulmonary colonization capacity of TGF-β1-depleted cells. This suggests that while free TGF-β1 and exosomal TGF-β1 might play parallel roles in driving tumor metastasis, only exosomal TGF-β1 can significantly drive pulmonary vascular niche remodeling at lower dosages, eventually bolstering the extravasation and colonization of TNBC cells in the lungs. Even as the TGF-β pathway has a definitive role in disease-associated biology and several strategies to interrupt TGF-β signaling have been developed and pushed clinically, their significant side effects hinder clinical adoption [34, 35]. One potential therapeutic avenue, which targets TGF-β activity while side stepping toxicity, lies in selectively inhibiting TGF-β subtypes that might be critical drivers in disease-associated processes. TGF-β1 has been reported to be ubiquitously present in a wide array of human tumors, making it the most pertinent subtype in the tumor context [34]. Our findings illustrate that exosomal TGF-β1 predominantly appears as LAP-TGF-β1 (around 90%), with exosomes from pulmonary metastatic foci showing heightened loading, primarily targeting the lungs. Given the minute yet potent nature of LAP-TGF-β1 in exosomes from pulmonary metastatic foci, strategically inhibiting the loading of tumor exosomal LAP-TGF-β1 presents itself as a pioneering strategy for treating the dissemination of pulmonary micrometastatic lesions.

**Fig. 9:**
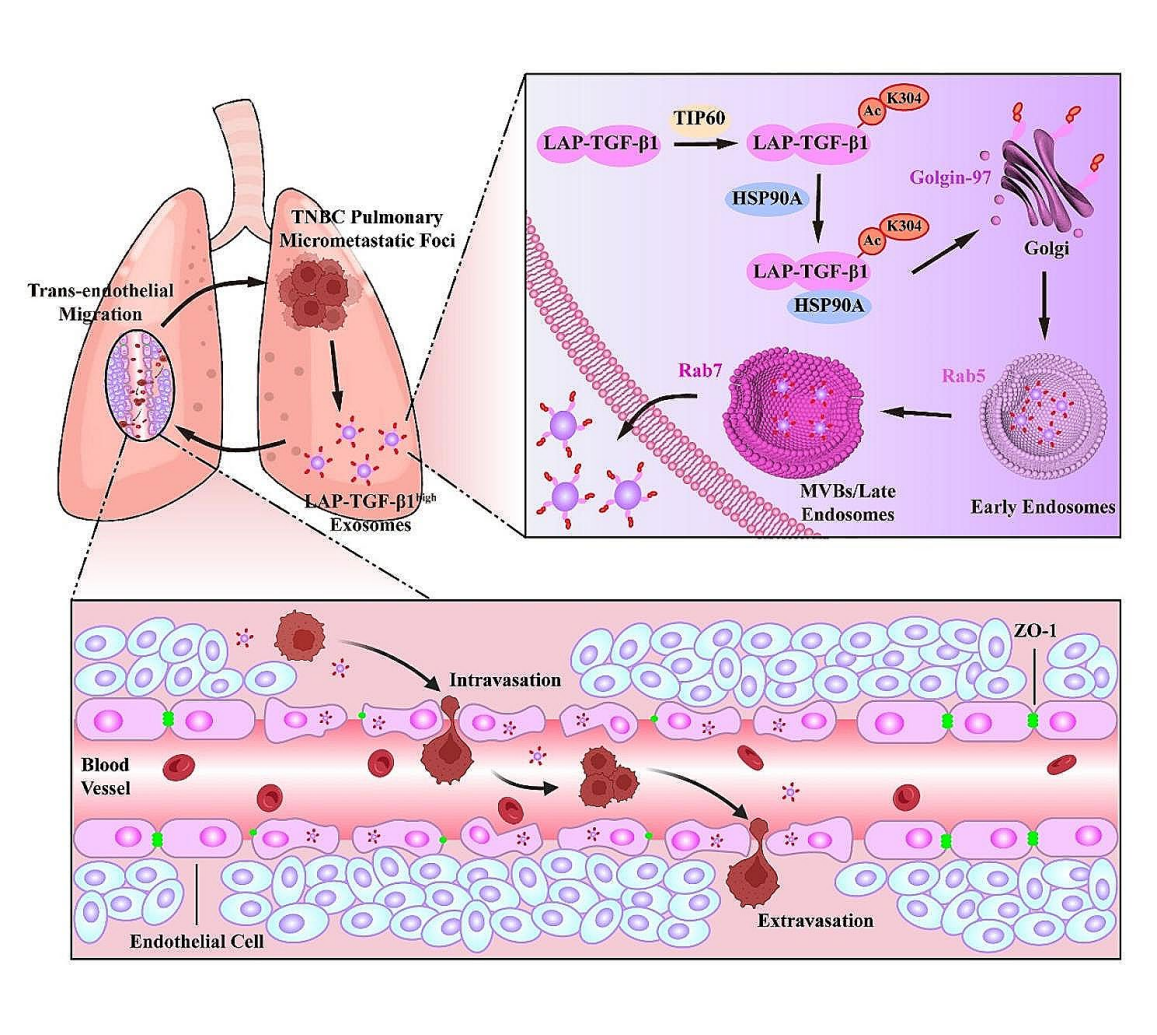
Schematic elucidation of LAP-TGF-β1 acetylation’s role in TNBC lung micro-metastases. LAP-TGF-β1 on exosomes from lung metastatic foci is acetylated at its K304 site via TIP60 mediation, leading to its interaction with HSP90A and facilitating its translocation from the Golgi apparatus to endosomes, resulting in the generation of exosomes with elevated LAP-TGF-β1 expression, reshaping the lung vascular niche, and ultimately promoting the dissemination of lung micro-metastases in TNBC

While numerous pathways participate in protein loading into exosomes [36], the mechanism underlying LAP-TGF-β1 transport into exosomes remains elusive. Previous research has pointed towards LAP-TGF-β1 binding to exosome surfaces via a heparin and chondroitin sulfate-sensitive glycoprotein interaction [24]. Yet, this fails to account for the elevated proportions of LAP-TGF-β1 in tumor cell exosomes, suggesting a unique tumor-centric exosomal loading pathway for LAP-TGF-β1. Recent discoveries have identified multiple cargo proteins, like IL-1β, LAMP2A, and the fusion protein RAB22A-NEOF1, that are transported into vesicles via chaperone proteins [39, 40, 62]. Although microvesicles and exosomes originate from different intracellular sites, their biogenesis often shares striking similarities, complicating the task of distinguishing these vesicular subgroups [63]. The location of cargo generation in the endocrine system offers subtle distinctions for extracellular vesicles: microvesicles on the plasma membrane and exosomes on the limiting membrane of multivesicular endosomes (MVE) [36]. Our research indicates that while HSP90A binds to LAP-TGF-β1 (the primary form of TGF-β1 in exosomes), it doesn’t promote LAP-TGF-β1’s cytoplasmic to plasma membrane transport, suggesting its entry into exosomes is through association with HSP90A. As a multifunctional peptide, LAP not only modulates critical developmental roles of TGF-β1 but also maintains tissue homeostasis in adulthood [64, 65]. TGF-β1 activation involves conformational changes in the N-terminus of LAP, conferred by its association with TGF-β1 during latency [66, 67]. The TGF-β1 domain of LAP-TGF-β1 interacts with HSP90A, and the LAP domain augments this association. Syntenin appears pivotal in exosome-mediated communication, linking membrane cargo to the ESCRT machinery through ALIX [68, 69]. Furthermore, studies have alluded to LAP-TGF-β1’s association with exosomal membranes and participation in endosomal signaling [24]. Our findings demonstrate that the interaction between LAP-TGF-β1 and HSP90A accelerates the transport of exosomal membrane cargo – facilitating the transfer of LAP-TGF-β1 from the Golgi to endosomes and its release from tumor cell exosomes (Fig. 9).

Furthermore, our work has shed light on the post-translational modifications of LAP-TGF-β1, which are requisite for its exosomal transport and subsequent facilitation of pulmonary micrometastasis dissemination. Contemporary studies illustrate that alterations in ubiquitination, phosphorylation, glycosylation, palmitoylation, and sumoylation facilitate the loading of specific proteins into multivesicular structures and tiny extracellular vesicles [70–73]. Multiple lines of research have established the link between dysregulated acetylation and an array of human diseases, encompassing immunity, inflammation, metabolism, and cancer [74]. We have, for the first time, pinpointed acetylation as vital for the exosomal loading of LAP-TGF-β1. Assisted by HSP90A, TIP60 activates acetylation of LAP-TGF-β1, driving its entry into exosomes, leading to pulmonary vascular niche remodeling, thereby furthering pulmonary micrometastasis dissemination. Moreover, through the acetylated non-canonical KFERQ-like sequence (300IDFRK304), LAP-TGF-β1 associates with HSP90A and is subsequently loaded into exosomes.

## Conclusions

In summation, our study has demystified the role of LAP-TGF-β1 in the dissemination of TNBC pulmonary micrometastasis and the underlying process of LAP-TGF-β1’s exosomal transport, thereby outlining a viable strategy for therapeutically targeting TNBC lung metastasis. Nonetheless, it’s pertinent to recognize that TGF-β1, being a pleiotropic cytokine, impacts the activities of various immune and non-immune cells. This possibly elucidates why TGF-β1 stands out as an appealing yet challenging therapeutic target for cancer, as multiple strategies modulating this cytokine have raised safety concerns. We have yet to delve into the potential impact of minute and potent pulmonary micrometastasis exosomal LAP-TGF-β1 on the activity of tumor-infiltrating immune cells. Additionally, whether curtailing exosomal LAP-TGF-β1 loading would hinder the dissemination of pulmonary micrometastasis in other cancer types remains an open question warranting further exploration.

### Electronic supplementary material

Below is the link to the electronic supplementary material.

Supplementary Material 1

Supplementary Material 2

## Data Availability

No datasets were generated or analysed during the current study.

## Abbreviations

***231 Parental***: MDA-MB-231 parental
***549 Parental***: BT-549 parental
***BLI***: bioluminescence imaging
***ELISA***: enzyme-linked immunosorbent assay
***EMT***: epithelial-mesenchymal transition
***ESCRT***: endosomal sorting complex required for transport
***Exo***: exosome
***GSEA***: gene set enrichment analysis
***HCl***: hydrochloric acid
***KEGG***: Kyoto Encyclopedia of Genes and Genomes
***KO***: knockout
***LAP***: latency-associated peptide
***LuECs***: lung endothelial cells
***MVBs***: multivesicular bodies
***MVE***: multivesicular endosomes
***NAM***: nicotinamide
***NTA***: nanoparticle tracking analysis
***TEM***: transmission electron microscopy
***TNBC***: triple-negative breast cancer
***TJs***: tight junctions
***TSA***: Trichostatin A
***WT***: wild-type
