# Supplementary material for: The incorporation of acetylated LAP-TGF-β1 proteins into exosomes promotes TNBC cell dissemination in lung micro-metastasis
Source: Mol Cancer. 2024 Apr 25;23:82. doi: 10.1186/s12943-024-01995-z (PMC11044330; doi:10.1186/s12943-024-01995-z)
Supplement: Supplementary file 1 — Supplementary Material 1 [file 12943_2024_1995_MOESM1_ESM.docx]

**The incorporation of acetylated LAP-TGF-β1 proteins into exosomes promotes TNBC cell dissemination in lung micro-metastasis**

Pei Yu^1†^, Yubao Han^1†^, Lulu Meng^1^, Zengying Tang^1^, Zhiwei Jin^1^, Zhenzhen Zhang^2^, Yunjiang Zhou^3^, Jun Luo^1^, Jianguang Luo^1^, Chao Han^1^, Chao Zhang^1*^, Lingyi Kong^1*^

^1^State Key Laboratory of Natural Medicines and Jiangsu Key Laboratory of Bioactive Natural Product Research, School of Traditional Chinese Pharmacy, China Pharmaceutical University, Nanjing 211198, China.

^2^Institute of Veterinary Science, Jiangsu Academy of Agricultural Sciences, Nanjing 210014, China.

^3^State Key Laboratory of Natural Medicines, School of Basic Medicine and Clinical Pharmacy, China Pharmaceutical University, Nanjing 211198, China.

^*^ Corresponding authors.

E-mail: cpu_lykong@126.com (Lingyi Kong), zhangchao@cpu.edu.cn (Chao Zhang)

^†^ These authors contributed equally to this work.

**Supplementary Materials and methods**

Cell lines and transfection procedures

The human cell lines MDA-MB-231 (referred to as 231 Parental), BT-549 (referred to as 549 Parental), HOS, and A549 were sourced from the Cell Bank of the Shanghai Institute of Biochemistry and Cell Biology, Chinese Academy of Sciences, Shanghai, China. These cells were cultured in either DMEM or F-12K medium (Gibco), with both media enriched with 10% fetal bovine serum from Gemini Bio-Products, CA, USA. These cultures were maintained in a humidified incubator set at 37°C, with an atmosphere of 5% CO_2_. Regular examinations ensured that the cell lines were free from mycoplasma contamination.

For the generation of stable cell lines, cells approaching 70% confluence were infected with HBLV-GFP-Puro-Luc lentivirus, provided by Hanbio Biotechnology, Shanghai, China. This infection process was conducted in complete media enhanced with 10 μg/mL of polybrene from Hanbio Biotechnology. Post-infection, puromycin (Yeasen Biotechnology, Shanghai, China) was employed for the selection of successfully infected cells.

Primary culture of lung-tropic cells

Lung-tropic cells were established by injecting 5 × 10^5^ units of HOS^GFP-Luc^, 231 Parental^GFP-Luc^, 549 Parental^GFP-Luc^, or 231 Parental^CD63-pEGFP^ cells intravenously into NCG-HLA-A2.1 mice (aged between 6-8 weeks, GemPharmatech, Nanjing, China). Six weeks post-injection, the mice were euthanized, and lung tissues were harvested. The lungs were digested at 37°C with 1 mg/mL collagenase I and hyaluronidase (both from Sigma-Aldrich, Shanghai, China) for one hour, followed by erythrocyte lysis in a lysis buffer (Beyotime, Shanghai, China) at 4°C for ten minutes. After thorough washing with PBS, cells were cultured in dishes until they reached enough for sorting GFP-positive cells via flow cytometry (BD Biosciences, New York, USA), yielding HOS LuT1^GFP-Luc^, 231 LuT1^GFP-Luc^, 231 LuT1^CD63-pEGFP^, and 549 LuT1^GFP-Luc^ cells. Subsequently, 5 × 10^5^ of either HOS LuT1^GFP-Luc^, 231 LuT1^GFP-Luc^, 231 LuT1^CD63-pEGFP^, or 549 LuT1^GFP-Luc^ cells were washed in PBS, collected, and reinjected into mice in a volume of 0.1 ml via tail vein. Five weeks post-injection, the mice were euthanized to harvest the lungs, obtaining HOS LuT2^GFP-Luc^, 231 LuT2^GFP-Luc^, 549 LuT2^GFP-Luc^, and 231 LuT2^CD63-pEGFP^ cells. Finally, 5 × 10^5^ HOS LuT2^GFP-Luc^, 231 LuT2^GFP-Luc^, 549 LuT2^GFP-Luc^, and 231 LuT2^CD63-pEGFP^ cells were again injected into mice. After four weeks, the mice were euthanized, and their lungs were harvested to obtain HOS LuT3^GFP-Luc^, 231 LuT3^GFP-Luc^, 549 LuT3^GFP-Luc^, or 231 LuT3^CD63-pEGFP^ cells.

Real-time Quantitative PCR

Upon reaching 50% confluence, 231 LuT3 cells were transfected with corresponding siRNAs (10 µL/2.5 nM) and incubated for 48 hours. Total RNA was then extracted utilizing the RNA-Quick Purification kit from ESscience, Shanghai, China. This RNA was subsequently reverse transcribed into cDNA with a kit from Vazyme, Nanjing, China. The quantitative real-time PCR procedure utilized SYBR Green Master Mix from Vazyme, and amplification was monitored using a LightCycler 480 Detector (Roche, Mannheim, Germany). Specific primers for this process are listed in Supplementary Table 2.

Cell migration assays

Migration assays employed Transwell Permeable Supports with 8 µm pores (Corning, NY, USA) as previously described [1]. GFP-labeled cells were introduced (5x10^4^ cells/well) into the upper chamber containing serum-free DMEM. Post 24-hour incubation, non-migrated cells on the top surface were removed, and the transwell membranes were scanned for migrated GFP^+^ cells using an inverted fluorescence microscope (Nikon, Tokyo, Japan). Migrated cell counts were conducted manually.

Isolation and characterization of lung endothelial cells (LuECs)

As per previous protocols [2], mouse lung tissues were harvested, minced, and digested with 1 mg/ml collagenase I for 1 hour. Following straining through a 70μm filter, cells underwent sequential magnetic sorting: first using CD31-conjugated dynabeads and then ICAM2-conjugated dynabeads. LuEC purity was validated by the expression of VE-cadherin and CD31, and by the absence of CD45.

Angiogenesis assay

LuECs were cultured in F12K medium enriched with 20% exosome-free serum, 1% non-essential amino acids (Thermo Scientific), 0.05 mg/mL endothelial cell growth supplement (Sigma-Aldrich), 20 mM HEPES, and 0.1 mg/mL heparin. Specific in vitro angiogenesis assay protocols and analyses were as mentioned earlier [3, 4].

Endothelial permeability assessment

LuEC monolayer permeability on transwell filters (0.4-µm pore size; Corning) was quantified utilizing rhodamine B isothiocyanate-dextran (average MW 70,000; Sigma). Briefly, the top chamber was supplemented with 20 mg/mL rhodamine-dextran, and the fluorescent diffusion into the bottom chamber was monitored over time on a SpectraMax microplate reader (Molecular Devices, CA, USA) at specified excitation and emission wavelengths.

Western blot analysis

Cellular and exosomal proteins were extracted using RIPA buffer supplemented with a phosphatase/protease inhibitor cocktail (Selleck, Texas, USA). Following previously cited protocols [5], protein complexes were visualized on the ChemiDOC™ system (Bio-Rad Laboratories, Hercules, CA). Specific antibodies are delineated in Supplementary Table 3.

Immunoprecipitation and immunoblotting

Upon reaching 80-90% confluence in 10-cm dishes, 231 parental or 231 LuT3 cells were transfected with 10 µg of specific plasmids and incubated for 48 hours. Procedures were followed as previously detailed [5]. The utilized antibodies are enumerated in Supplementary Table 2. TSA (1 μM) and NAM (10 mM) were procured from CSNpharm (Chicago, USA), both with purities exceeding 98%.

*In vitro* metastasis assay

To model metastasis, BD BioCoat Matrigel Invasion 24-well Chambers with 8 μm pores (Corning) were first rehydrated using serum-free media for 2 hours at 37°C. LuECs pretreated with LuEC medium (5x10^4^ per well) were subsequently seeded into these chambers. After a 24-hour period, the lower chambers were overlaid with 300 μL of Matrigel, followed by the addition of 500 μL DMEM supplemented with 10% FBS. A top this, GFP-labeled cells (2.5x10^4^ cells per well) were seeded in serum-free DMEM. Post incubation for 2-4 days in a CO_2_ chamber, the insert was carefully removed, rinsed in PBS, and fixed in 4% paraformaldehyde for 30 minutes. A Nikon fluorescent microscope facilitated the observation and enumeration of cancer cells that successfully migrated through the LuECs. To further monitor invasion, cancer cells breaching the Matrigel of the lower chamber were cultured in Matrigel-enriched media for an additional 5-8 days, aiming for mammosphere formation. Resultant tumor spheroids were visualized and quantified via fluorescence microscopy.

Immunofluorescence analysis

Following treatments, LuECs and 231 parental cells were washed with PBS, fixed in 4% paraformaldehyde for 30 minutes at room temperature, and then permeabilized with 0.5% Triton X-100 for 10 minutes. Non-specific binding was minimized by incubating cells in 5% BSA for an hour. Primary antibodies tailored to specific antigens were introduced and incubated overnight at 4°C. Subsequently, cells were exposed to either Alexa Fluor 594 or Alexa Fluor-488 conjugated secondary antibodies (Yeasen) for 2 hours at room temperature. Nuclei visualization was achieved using DAPI staining (Yeasen). High-resolution fluorescence images were captured using the ImageXpress® Micro Confocal system (Molecular Devices, Sunnyvale, California, USA), with further image processing carried out via MetaXpress software (Molecular Devices).

TGFB1 knockout (KO) generation

To engineer TGFB1-deficient 231 LuT3 cells, cells were transfected with the pCWCas9-containing plasmid (52961, Addgene) to facilitate CRISPR/Cas9-mediated genomic editing. The gRNA sequence specifically targeting the human TGFB1 gene (ACCAAAGCAGGGTTCACTAC) was incorporated into the PLX-sgRNA plasmid (50662, Addgene). Following a 48-hour post-transfection period, blasticidin selection was applied for two weeks to isolate successfully edited single clones. Western blotting assessed the extent of TGF-β1 protein knockdown, and successful gene editing was confirmed via detection of insertion/deletion mutations using Sanger sequencing.

Bioluminescence imaging

Post intraperitoneal administration of luciferin potassium (Yeasen Biotechnology), in vivo metastatic progression in the lungs was visualized using the Maestro™ Bioluminescence Imaging System (PerkinElmer). Quantification of bioluminescence was achieved via Living Image software, measuring total photon flux in the lungs. Histological assessment provided insights into lesion count and total metastatic area in lung sections, with metastatic clusters defined as aggregates of ten or more tumor cells. Detailed histological procedures can be referred to in earlier publications [4].

Flow cytometry

24 hours following the injection of PKH67-marked exosomes, lungs from female NCG-HLA-A2.1 mice were harvested and processed. Lung tissues underwent enzymatic digestion with type I collagenase and subsequent filtering through a 70μm strainer to obtain a single-cell dispersion. Exosome uptake by lung fibroblasts and endothelial cells was determined by their respective markers, S100A4^+^PKH67^+^ and CD31^+^PKH67^+^. Analysis and cell sorting were facilitated using a Beckman Coulter flow cytometer, with subsequent data interpretation via FlowJo 10.8.1.

Histological procedures

Standard procedures as detailed in prior studies were followed for tissue processing and immunostaining [6, 7]. For antibody specifics, readers are direc ted to Supplementary Table 3.

**References**

1. Chen C, Ma T, Zhang C, Zhang H, Bai L, Kong L, Luo J: Down-regulation of aquaporin 5-mediated epithelial-mesenchymal transition and anti-metastatic effect by natural product Cairicoside E in colorectal cancer. *Mol Carcinog* 2017, 56:2692-2705.

2. Wang J, Niu N, Xu S, Jin ZG: A simple protocol for isolating mouse lung endothelial cells. *Sci Rep* 2019, 9:1458.

3. Zhu J, Jin Z, Yang L, Zhao C, Hu J, Chen J, Han Y, Yu P, Luo J, Kong L, Zhang C: Ginkgolide B targets and inhibits creatine kinase B to regulate the CCT/TRiC-SK1 axis and exerts pro-angiogenic activity in middle cerebral artery occlusion mice. *Pharmacol Res* 2022, 180:106240.

4. Rodrigues G, Hoshino A, Kenific CM, Matei IR, Steiner L, Freitas D, Kim HS, Oxley PR, Scandariato I, Casanova-Salas I, et al: Tumour exosomal CEMIP protein promotes cancer cell colonization in brain metastasis. *Nat Cell Biol* 2019, 21:1403-1412.

5. Yu P, Zhu X, Zhu JL, Han YB, Kong LY: The Chk2-PKM2 axis promotes metabolic control of vasculogenic mimicry formation in p53-mutated triple-negative breast cancer. *Oncogene* 2021, 40:5262-5274.

6. Zhou W, Fong MY, Min Y, Somlo G, Liu L, Palomares MR, Yu Y, Chow A, O'Connor ST, Chin AR, et al: Cancer-secreted miR-105 destroys vascular endothelial barriers to promote metastasis. *Cancer Cell* 2014, 25:501-515.

7. Cheng X, Yu P, Zhou X, Zhu J, Han Y, Zhang C, Kong L: Enhanced tumor homing of pathogen-mimicking liposomes driven by R848 stimulation: A new platform for synergistic oncology therapy. *Acta Pharm Sin B* 2022, 12:924-938.

**Supplementary Figures and Figure Legends**

**
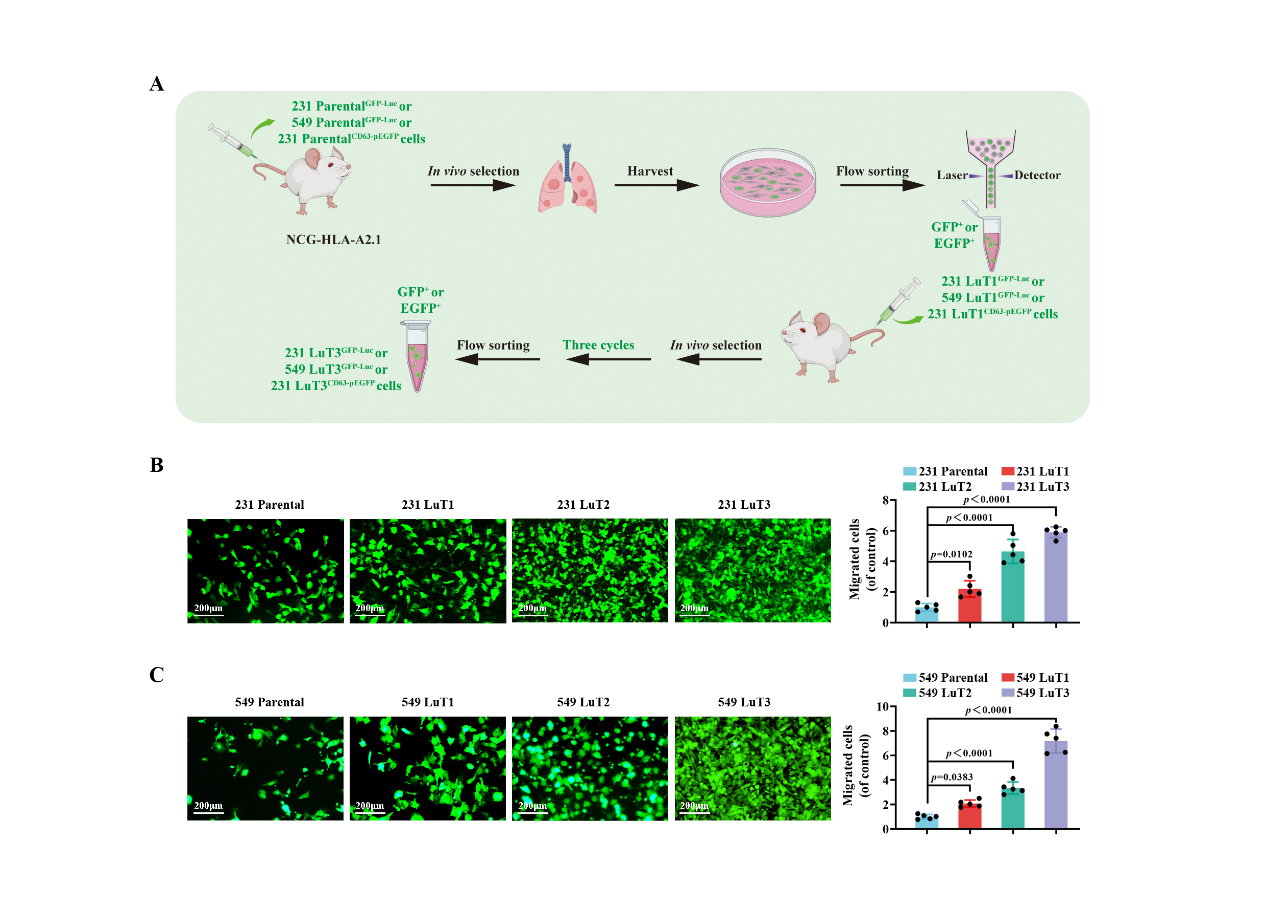
**

Fig. S1 Derivation of TNBC cells exhibiting enhanced lung metastatic potential. (A) Schematic representation of the *in vivo* approach to select TNBC cells highly inclined to metastasize to the lung. (B) Comparative transwell migration assays for 231 parental, 231 LuT1, 231 LuT2, and 231 LuT3 cells (*n*=5). (C) Comparative transwell migration assays for 549 parental, 549 LuT1, 549 LuT2, and 549 LuT3 cells (*n*=5). Scale bars: 200 μm. Data shown as means ± SD.


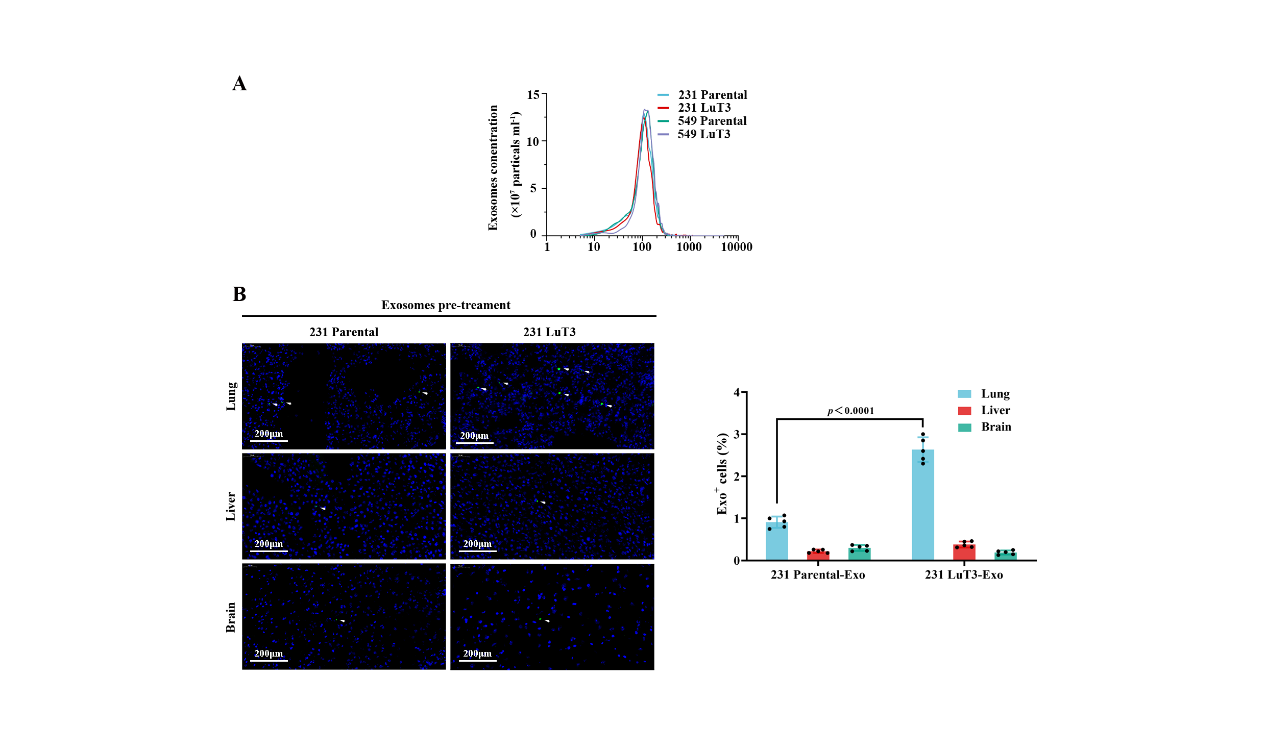


Fig. S2 Tissue-specific tracking of PKH67-tagged exosomes in NCG-HLA-A2.1 female mice. (A) Nanoparticle tracking analysis (NTA) of exosomes derived from 231 Parental, 231 LuT3, 549 Parental, and 231 LuT3 cells. (B) Depiction of PKH67-labeled exosome localization from 231 parental and 231 LuT3 cells in the lung, liver, and brain. Annotations mark exosome aggregations (*n*=5). Scale bars: 200 μm. Data shown as means ± SD.


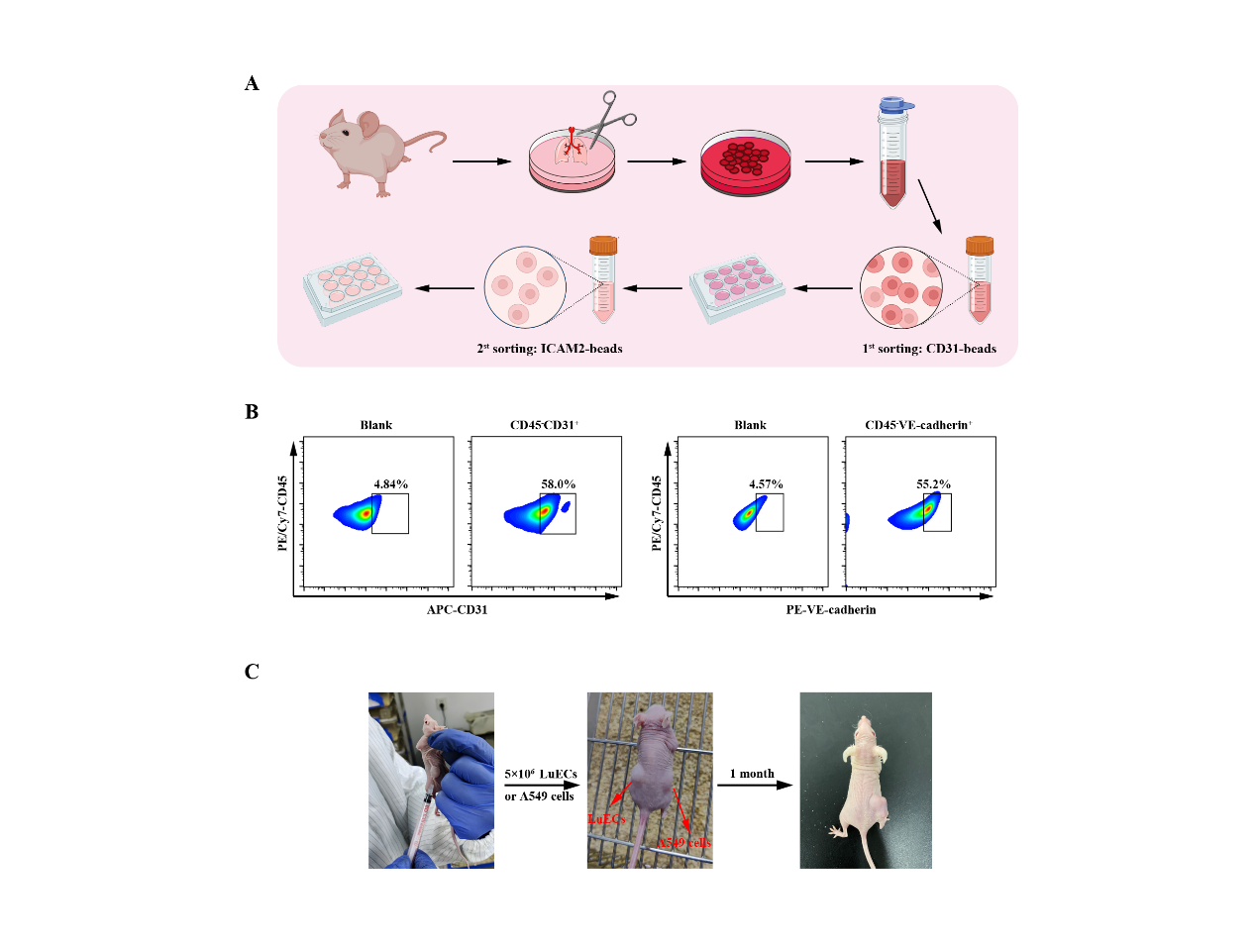


Fig. S3 Lung endothelial cells (LuECs): isolation and profiling. (A) A procedural illustration for LuEC extraction. (B) Isolation efficacy of LuECs assessed via flow cytometry. (C) Subcutaneous A549 cell and LuEC co-injection diagram.


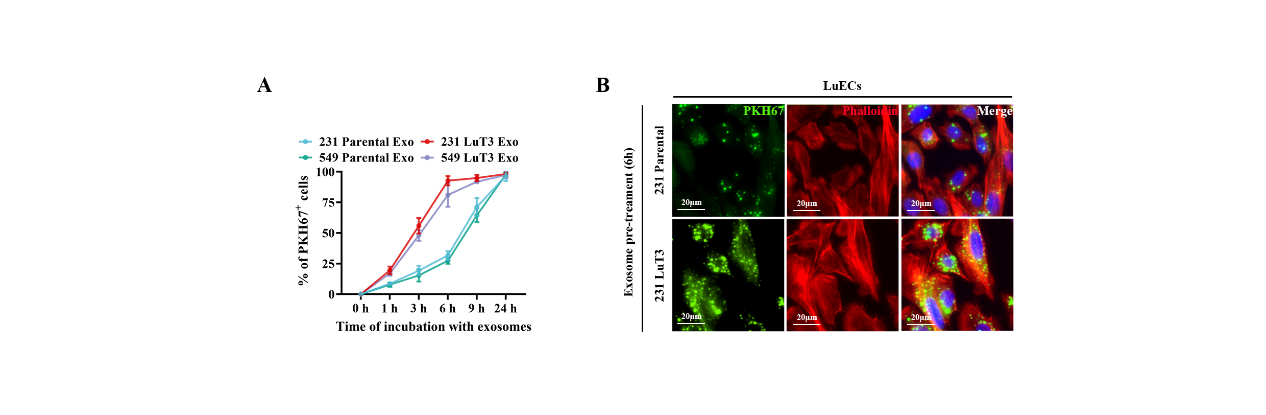


Fig. S4 Exosome assimilation by LuECs originating from 231 Parental and 231 LuT3 cells. (A) Flow cytometry analysis of LuECs for uptake of different PKH67-labeled exosomes at specified time points (*n*=3). (B) LuEC treatment with PKH67-tagged exosomes (green) for 6 hours, counterstained using Phalloidin (red) and DAPI (blue). Scale bars: 20 μm. Data shown as means ± SD.


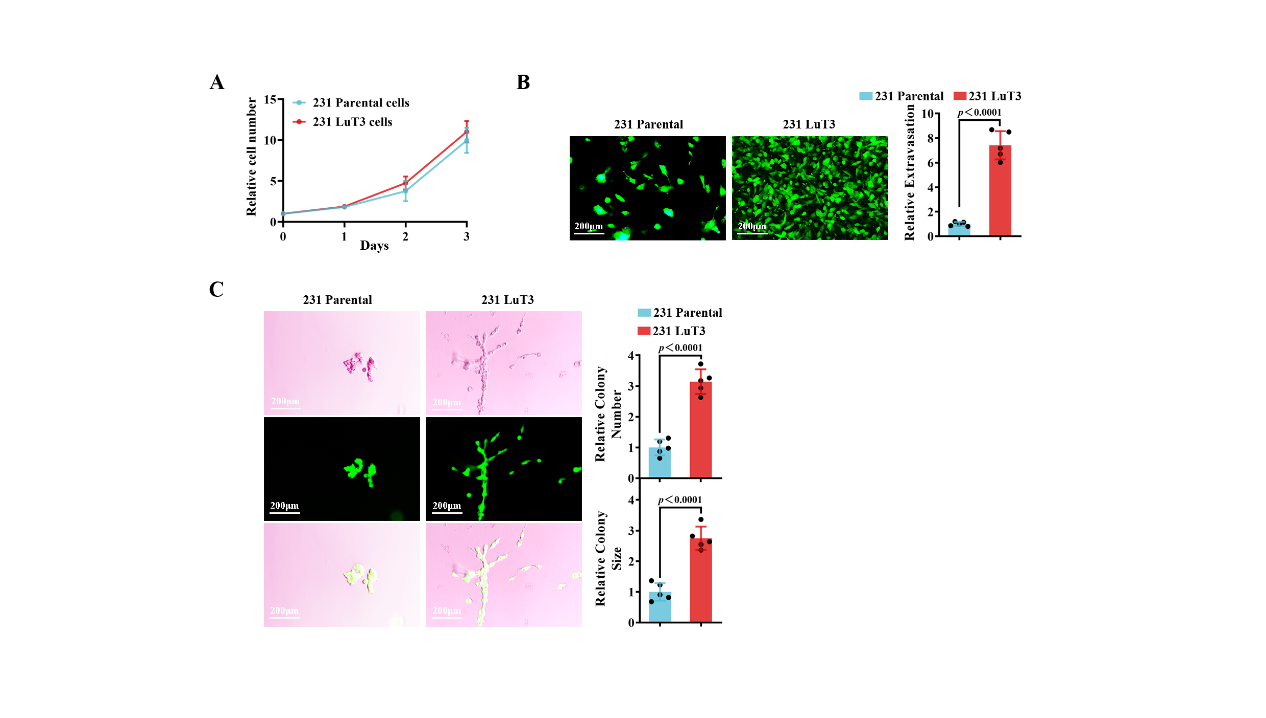


Fig. S5 *In vitro* assessment of lung metastasis by 231 Parental and 231 LuT3 cells. (A) Three-day proliferation curve comparing 231 parental and 231 LuT3 cells (*n*=3). (B-C) GFP-labeled 231 parental or 231 LuT3 cells were layered onto LuECs pre-incubated in insert chambers for 24 hours (*n*=5). Scale bars: 200 μm. (B) Evaluating extravasation at day 4. (C) Observation of invasion via the basement membrane, leading to mammosphere formation by day 8. Data shown as means ± SD.


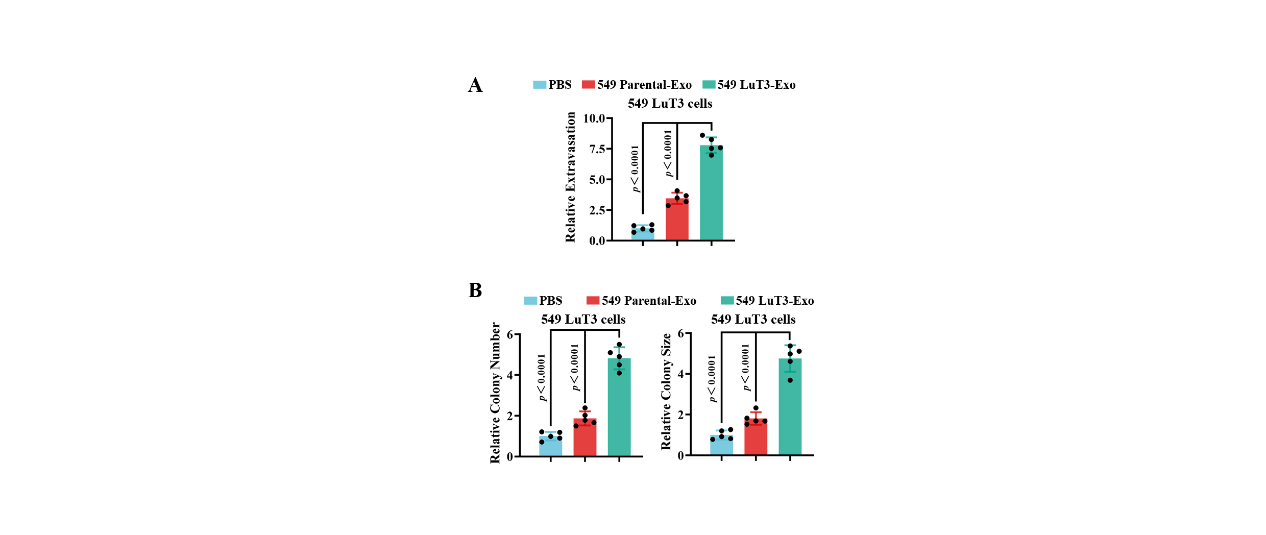


Fig. S6 Impact of exosomes on 549 LuT3 cell metastatic activity *in vitro*. (A-B) After treating LuECs with PBS or 549-derived exosomes for 24 hours, GFP-labeled 549 LuT3 cells were implanted into transwell inserts (*n*=5). Scale bars: 200 μm. (A) Extravasation (3 days). (B) Invasion through the basement membrane, followed by the development of a mammosphere (7 days). Data shown as means ± SD.


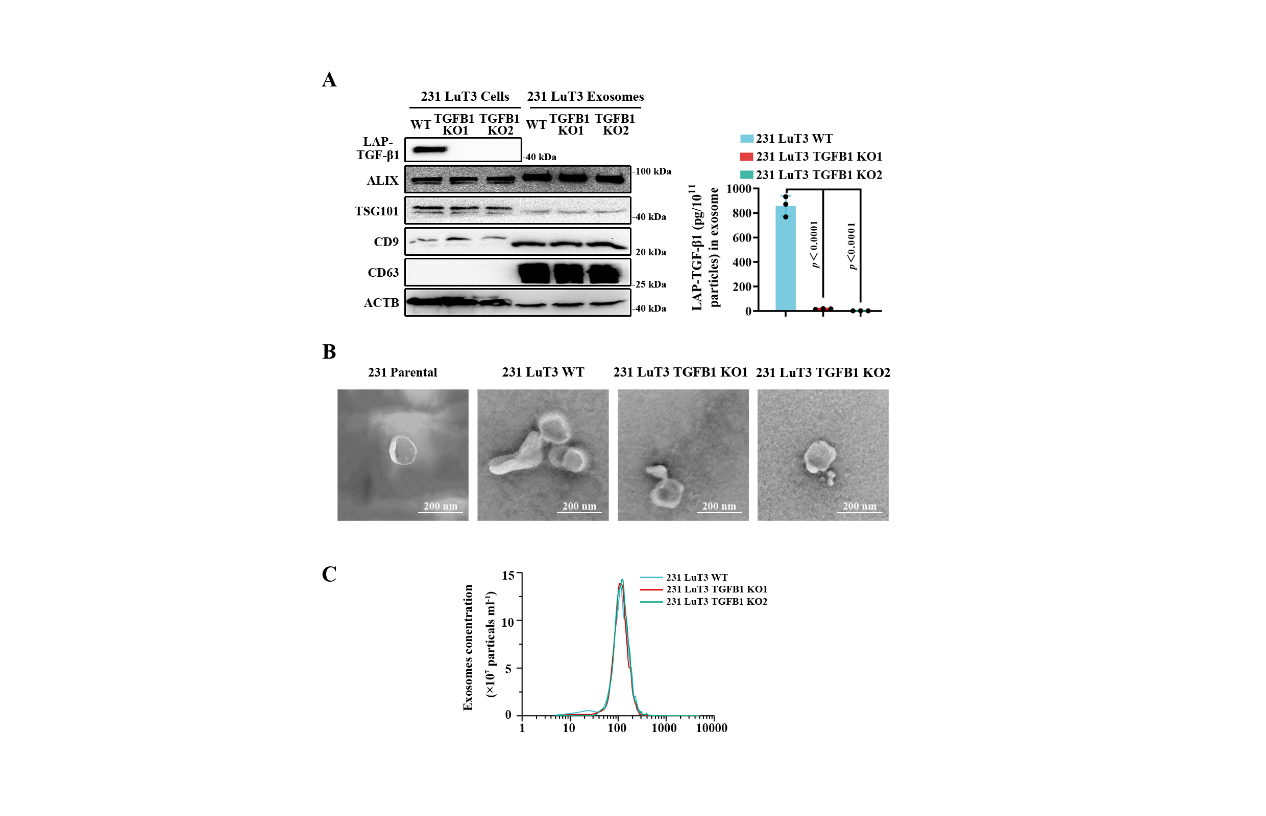


Fig. S7 Exosomal profiling of TNBC cell lines. (A) Comparative analysis of LAP-TGF-β1 expression alongside exosomal markers (including ALIX, TSG101, CD9, and CD63) between 231 LuT3 and 231 LuT3 TGFB1 KO cell lineages (*n*=3). (B) TEM and (C) NTA depicting exosomes derived from 231 parental, 231 LuT3, and 231 LuT3 TGFB1 KO cells. Data shown as means ± SD.

**
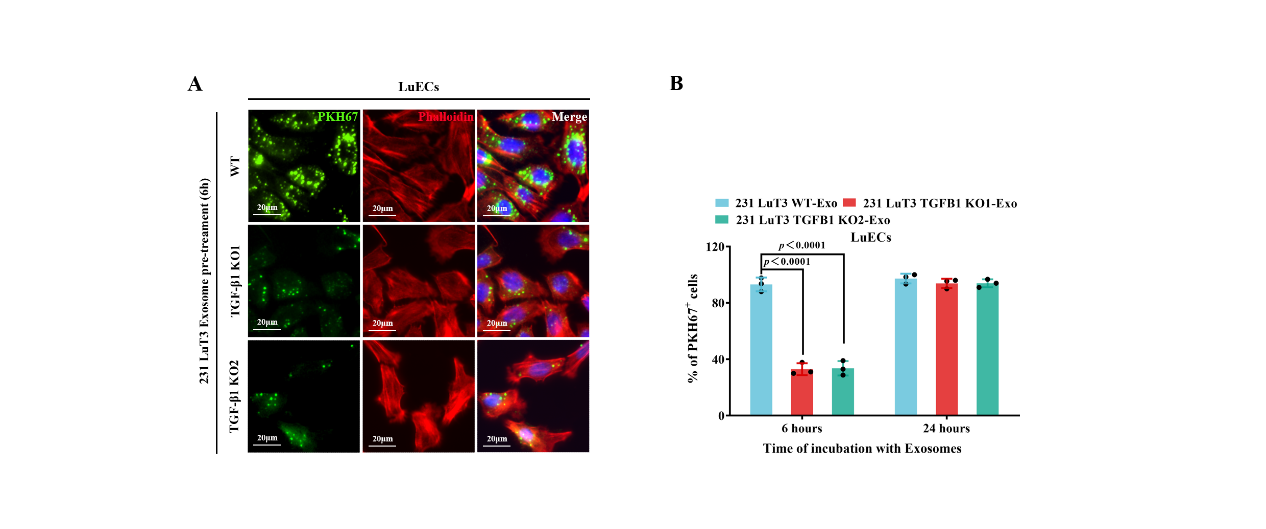
**

Fig. S8 Endocytosis of exosomes from 231 LuT3 and 231 LuT3 TGFB1 KO cells by LuECs. (A) LuECs treated with PKH67-labeled exosomes (green) for a 6-hour duration, counterstained using Phalloidin (red) and DAPI (blue). Scale bars: 20 μm. (B) Flow cytometric evaluation of PKH67 incorporation into LuECs following 6 and 24-hour exposure to PKH67-tagged exosomes (*n*=3). Data shown as means ± SD.

**
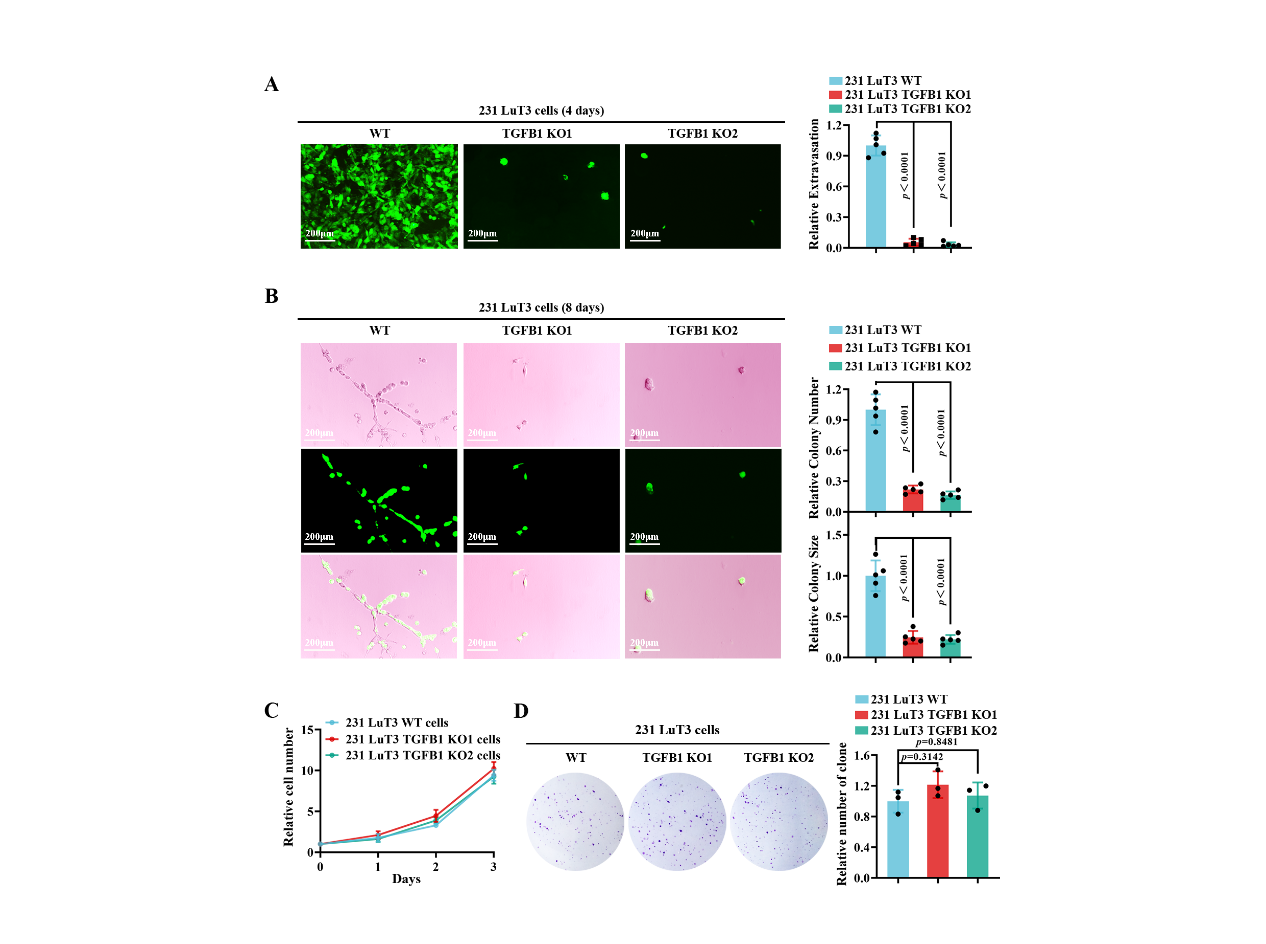
**

Fig. S9 *In vitro* examination of metastatic potential of 231 LuT3 and 231 LuT3 TGFB1 KO cells. (A-B) LuECs, post 24-hour seeding in chambers, were overlaid with either 231 LuT3 or 231 LuT3 TGFB1 KO cells (*n*=5). Scale bars: 200 μm. (A) Extravasation observed at day 4. (B) Basement membrane penetration leading to mammosphere formation by day 8. (C) Proliferative analysis of 231 LuT3 and 231 LuT3 TGFB1 KO cells over a span of three days (*n*=3). (D) Colony-forming potential and quantitative analysis of 231 LuT3 and 231 LuT3 TGFB1 KO cells at day 7 (*n*=3). Data shown as means ± SD.

**
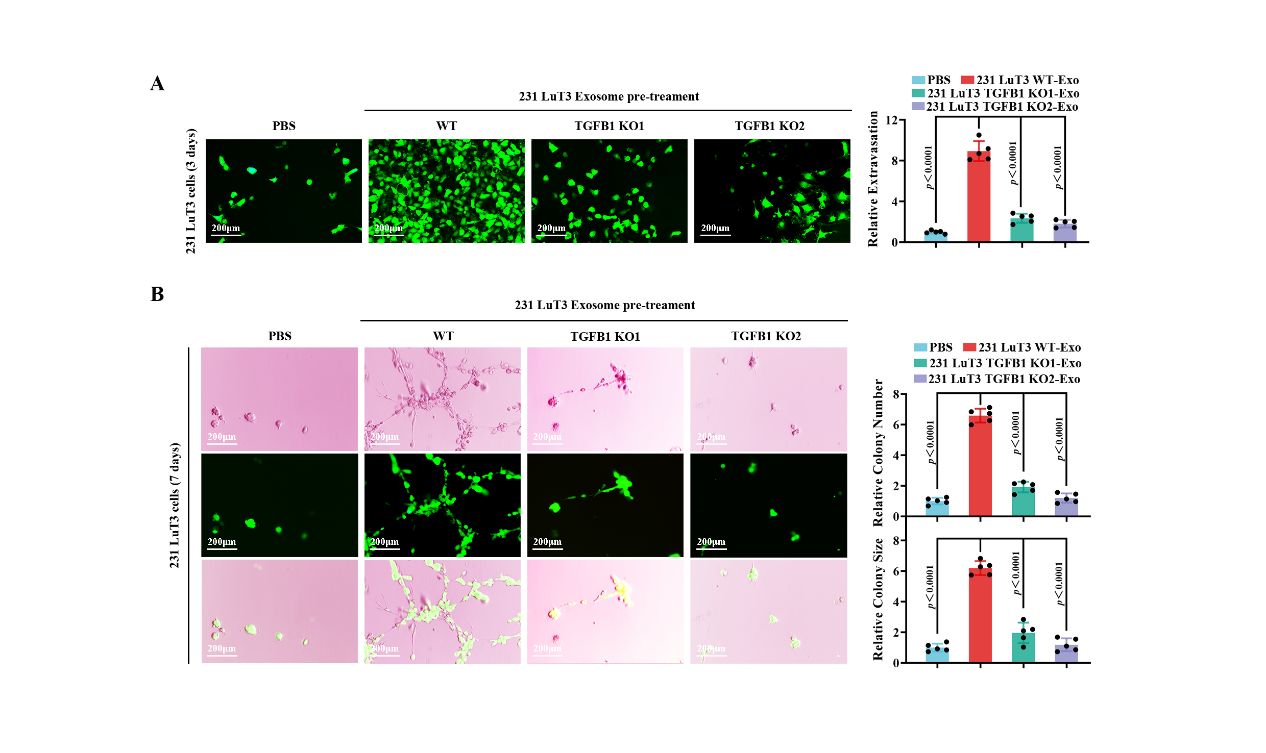
**

Fig. S10 Impact of exosomes on 231 LuT3 cell metastatic activity *in vitro*. (A-B) After treating LuECs with PBS or exosomes for 24 hours, GFP-labeled 231 LuT3 cells were introduced into transwell inserts (*n*=5). Scale bars: 200 μm. (A) Day 3, extravasation study. (B) Basement membrane breach followed by mammosphere emergence on day 7. Data shown as means ± SD.

**
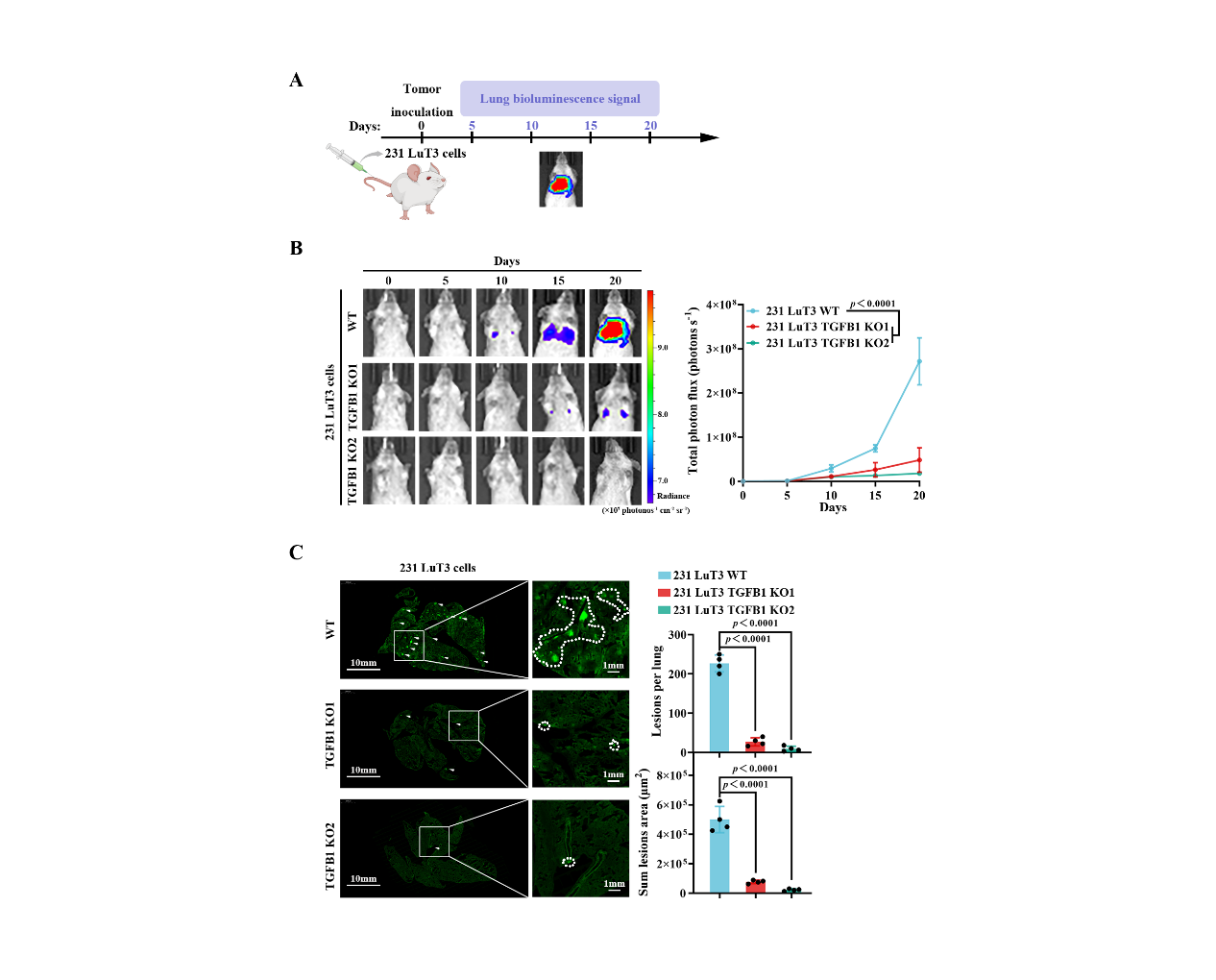
**

Fig. S11 TGF-β1 augmentation of lung micro-metastasis *in vivo*. (A-C) NCG-HLA-A2.1 mice received intravenous injections of GFP-Luc-tagged 231 LuT3 and 231 LuT3 TGFB1 KO cells (*n*=4). (A) Schematic overview of the TNBC lung metastasis mouse model. (B) On days 5, 10, 15, and 20 post-cell infusion, bioluminescence lung imaging alongside metastatic count is presented. (C) Lung sections at day 20 reveal GFP-marked metastasis (left images). On the right, comprehensive metastatic lung lesion metrics are provided. Scale bars: 10 mm and 1 mm. Data shown as means ± SD.


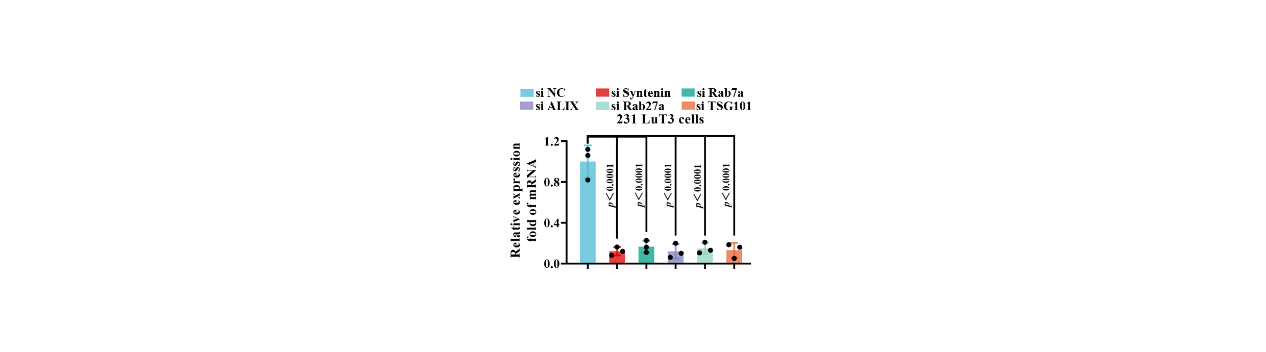


Fig. S12 Gene expression correlation with exosome genesis, movement, and discharge. Utilizing RT-qPCR, genes of interest were evaluated in 231 LuT3 cells post-transfection with si Syntenin, si Rab7a, si ALIX, si Rab27a, or si TSG101 (*n*=3). Data shown as means ± SD.


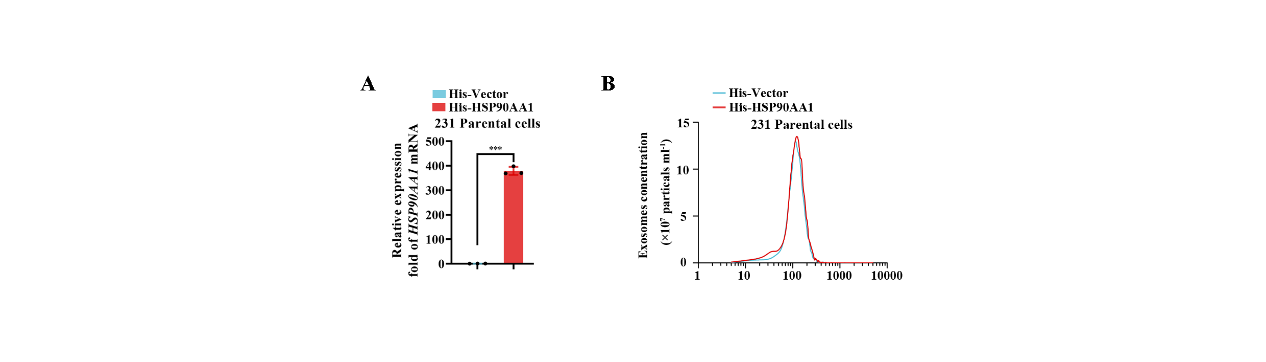


Fig. S13 Gene profiling of HSP90AA1-enhanced cells and their exosome NTA. (A) RT-qPCR quantification of HSP90AA1 in 231 parental cells post-transfection with either His-HSP90AA1 or his-vector (*n*=3). (B) Exosome NTA post the aforementioned transfections. Data shown as means ± SD.


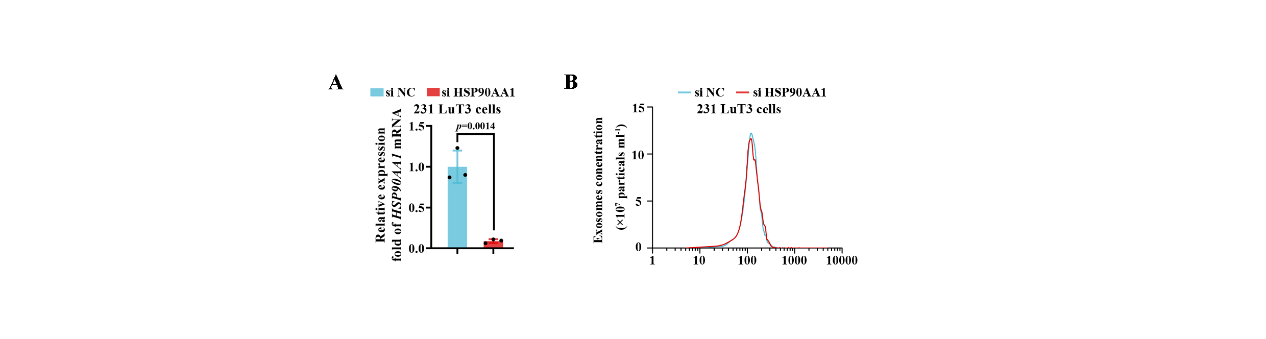


Fig. S14 Impact of HSP90AA1 suppression on gene expression and exosome NTA. (A) RT-qPCR-based HSP90AA1 assessment in 231 LuT3 cells post-transfection with either si HSP90AA1 or si NC (*n*=3). (B) Exosome NTA following the highlighted transfections. Data shown as means ± SD.


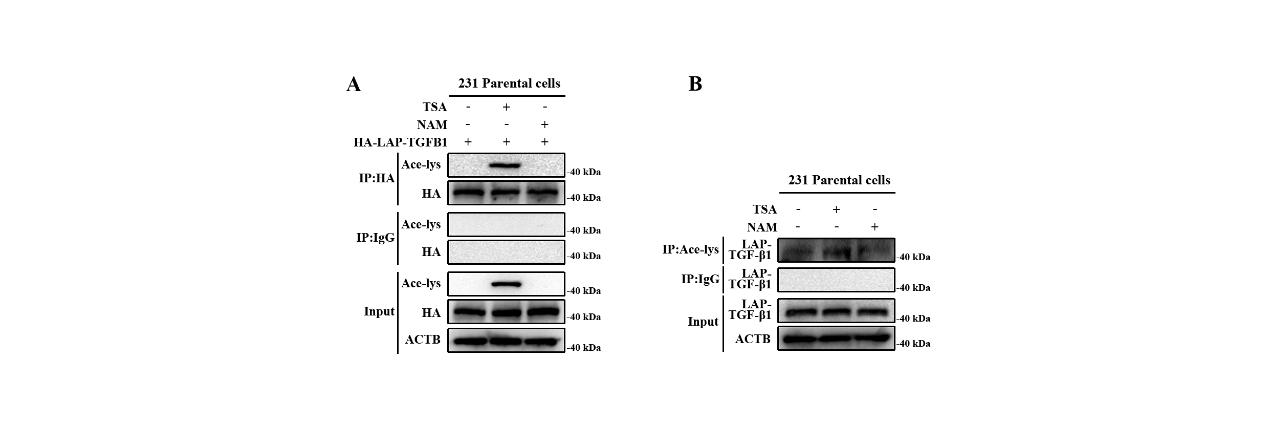


Fig. S15 LAP-TGF-β1 acetylation characterization. (**A**) 231 parental cells, after transfection with HA-LAP-TGFB1 and subsequent treatment with 1 μM TSA or 10 mM NAM, underwent purification using anti-HA agarose beads, followed by immunoprecipitation and immunoblotting using selected antibodies. (**B**) Post-treatment of 231 parental cells with either TSA or NAM, cell extracts were purified using anti-Ace-lys agarose beads. The extracts were then processed as described in (A).


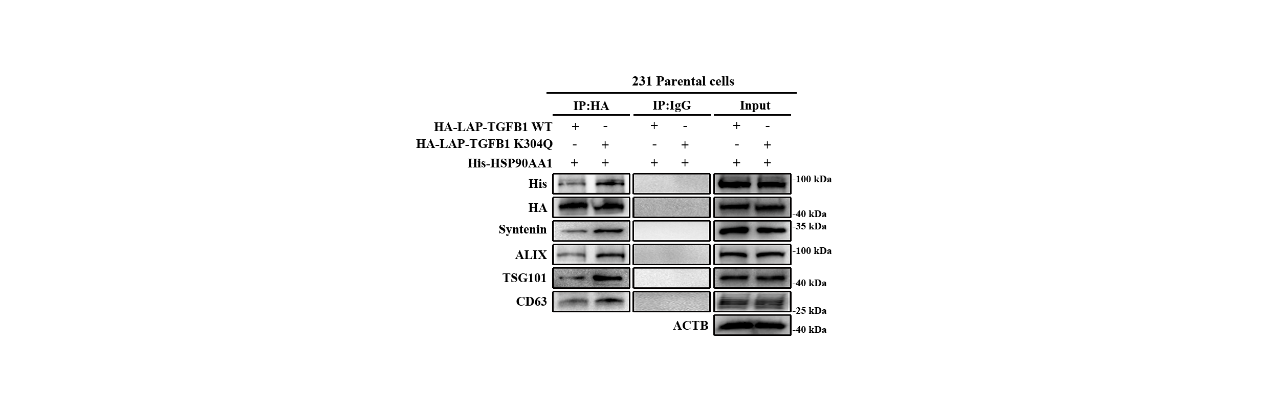


Fig. S16 K304 acetylation of LAP-TGF-β1 enhances affinity with HSP90A and related exosomal proteins. 231 parental cells were purified post co-transfection with His-HSP90AA1 and either HA-LAP-TGFB1 WT or HA-LAP-TGFB1 K304Q. The immunoprecipitated lysates were immunoblotted with the pertinent antibodies.


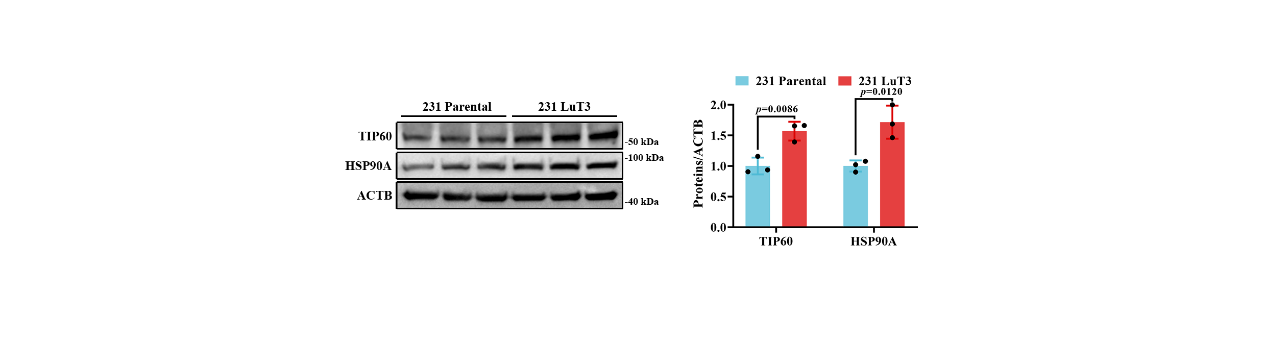


Fig. S17 Expression profile of HSP90A and TIP60 in tumor samples. GFP-Luc labeled 231 parental or 231 LuT3 cells were inoculated into the fourth mammary fat pad of NCG-HLA-A2.1 mice. Seventeen days post-injection, tumors were harvested (*n*=5). Western blotting was employed to evaluate HSP90A and TIP60 protein levels in these tumor specimens (*n*=3). Data shown as means ± SD.


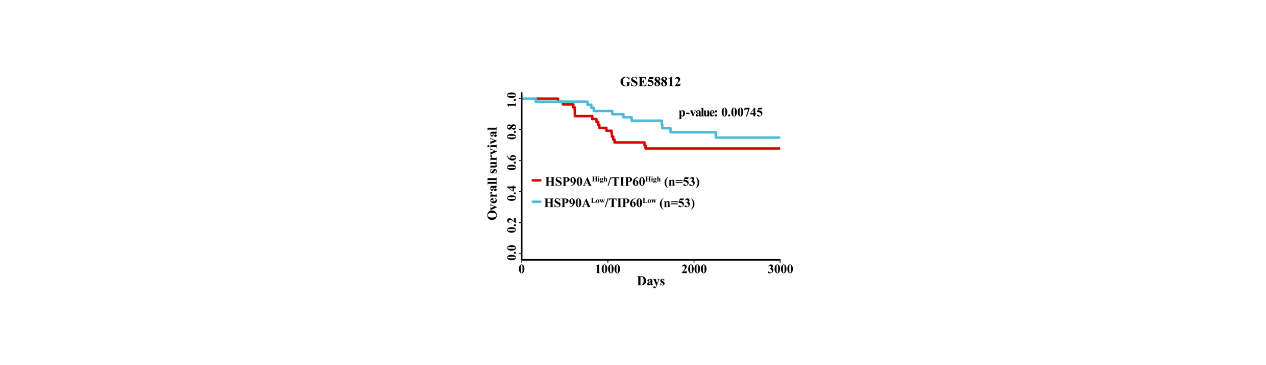


Fig. S18 Survival assessment in relation to HSP90A and TIP60 expression in TNBC cases. The Kaplan-Meier curve portrays overall survival based on HSP90A and TIP60 expression profiles in TNBC patients, utilizing the GSE58812 dataset (*n*=106).


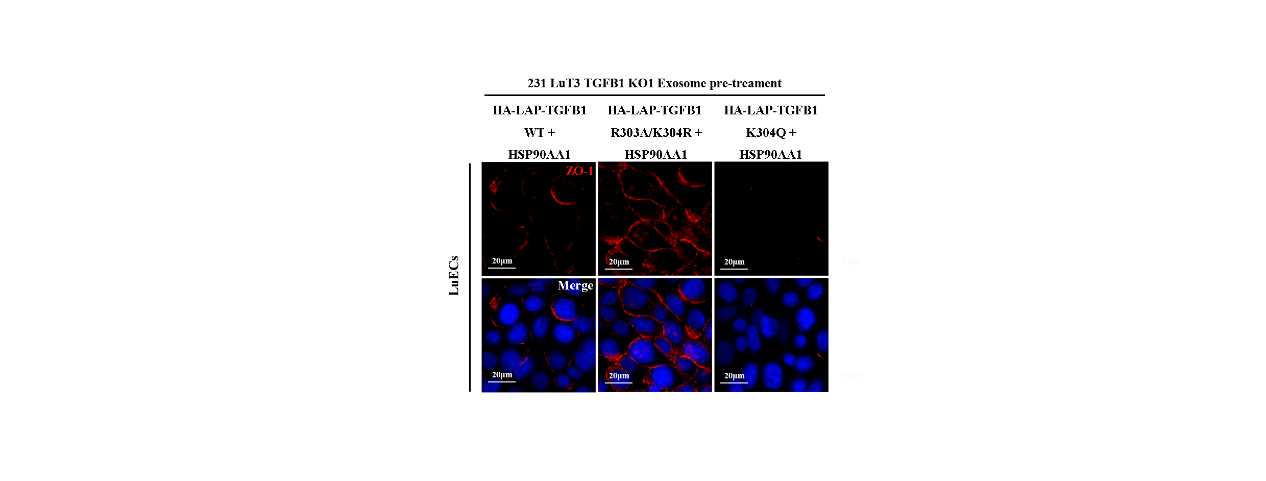


Fig. S19 Influence of acetylation status of LAP-TGF-β1 K304 residue on ZO-1 expression in LuECs via exosomal modulation. LuECs, post 24-hour exposure to exosomes derived from 231 LuT3 TGFB1 KO1 cells cotransfected with His-HSP90AA1 in combination with HA-LAP-TGFB1 WT, HA-LAP-TGFB1 R303A/K304A, or HA-LAP-TGFB1 K304Q, displayed ZO-1 (red) expression as detected through immunofluorescence. Scale bars: 20 μm.


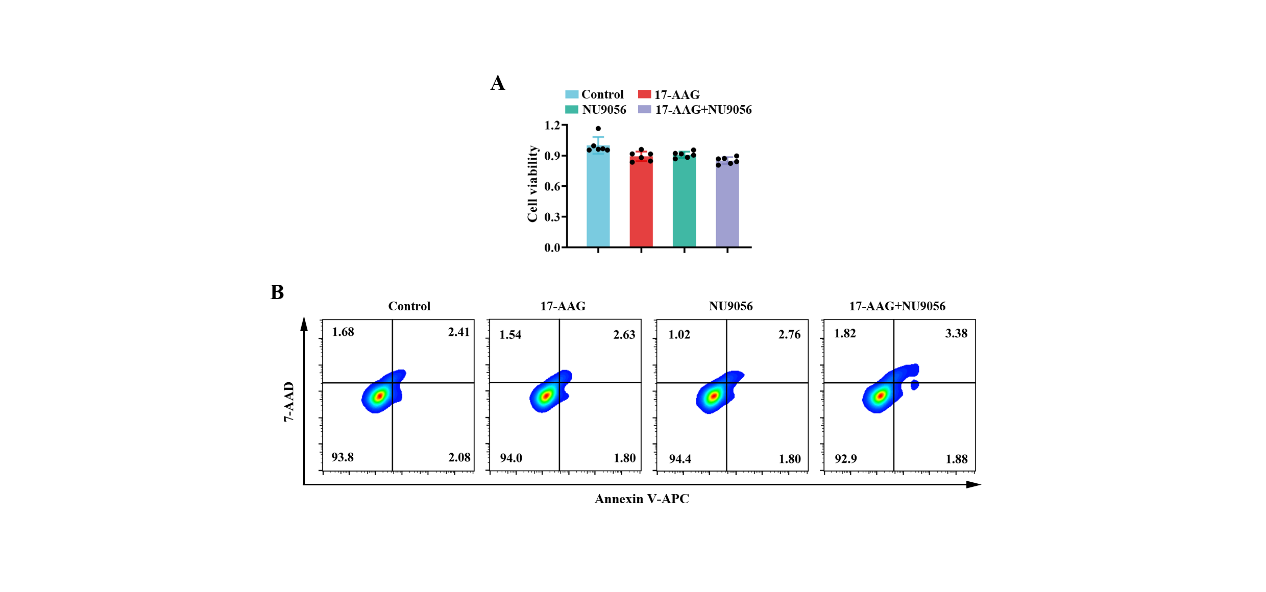


Fig. S20 Pharmacological inhibition of HSP90A and TIP60: impacts on cell viability and induced apoptosis. (A) Cell viability metric for 231 LuT3 cells (*n*=6). (B) Apoptotic assay outcomes in 231 LuT3 cells. Data shown as means ± SD.

**Supplementary Table 1 Sequences of siRNA primers**

| Gene name |  | Primer sequence (5’→3’) |
| --- | --- | --- |
| si Syntenin | F | CCUAUCCCUCACGAUGGAAAUTT |
|  | R | AUUUCCAUCGUGAGGGAUAGGTT |
| si HSP90AA1 | F | UACUUGGAGGAACGAAGAAUATT |
|  | R | UAUUCUUCGUUCCUCCAAGUATT |
| si Rab7a | F | GGCUAGUCACAAUGCAGAUAUTT |
|  | R | AUAUCUGCAUUGUGACUAGCCTT |
| si ALIX | F | CCAGAACAAAUGCAGUGAUAUTT |
|  | R | AUAUCACUGCAUUUGUUCUGGTT |
| si TSG101 | F | GCCUUAUAGAGGUAAUACAUATT |
|  | R | UAUGUAUUACCUCUAUAAGGCTT |
| si Rab27a | F | CCAGUGUACUUUACCAAUAUATT |
|  | R | UAUAUUGGUAAAGUACACUGGTT |

**Supplementary Table 2 Sequences of real time PCR primers**

| Gene name |  | Primer sequence (5’→3’) |
| --- | --- | --- |
| Syntenin | F | CTGCTCCTATCCCTCACGATG |
|  | R | GGCCACATTTGCACGTATTTCT |
| HSP90AA1 | F | AGGAGGTTGAGACGTTCGC |
|  | R | AGAGTTCGATCTTGTTTGTTCGG |
| Rab7a | F | GTGTTGCTGAAGGTTATCATCCT |
|  | R | GCTCCTATTGTGGCTTTGTACTG |
| ALIX | F | ATGGCGACATTCATCTCGGTG |
|  | R | CGCTTGGGTAAGTCTGCTGG |
| TSG101 | F | GAGAGCCAGCTCAAGAAAATGG |
|  | R | TGAGGTTCATTAGTTCCCTGGA |
| Rab27a | F | GCTTTGGGAGACTCTGGTGTA |
|  | R | TCAATGCCCACTGTTGTGATAAA |
| GAPDH | F | GAAAGCCTGCCGGTGACTAA |
|  | R | AGGAAAAGCATCACCCGGAG |

**Supplementary Table 3 Antibodies used in this study.**

| **Antigens** | **Lot, manufacturers** | **Application** |
| --- | --- | --- |
| CD63 | ab134045, Abcam | 1:5000 for WB |
| CD9 | ab92726, Abcam | 1:5000 for WB |
| TSG101 | ab125011, Abcam | 1:5000 for WB |
| ALIX | ab186429, Abcam | 1:5000 for WB |
| ACTB | 66009-1-lg, Proteintech | 1:20000 for WB |
| TGF-β1 | ab179695, Abcam | 1:1000 for WB |
| Syntenin | ab133267, Abcam | 1:5000 for WB |
| HSP90 | sc-101494, Santa Cruz | 1:200 for WB |
| Ac-lysine | sc-32268, Santa Cruz | 1:200 for WB  1:20 for IP |
| HA | 51064-2-AP, Proteintech | 1:5000 for WB  1:100 for IP |
| HSP90A | sc-13119, Santa Cruz | 1:1000 for WB |
| HA (HRP Conjugated) | M20021, Abmart | 1:100 for IP |
| flag | 66008-3-lg, Proteintech | 1:1000 for WB  1:50 for IP |
| His | 66005-1-Ig, Proteintech | 1:5000 for WB  1:100 for IP |
| GORASP2 | 10598-1-AP, Proteintech | 1:1000 for WB |
| LC3B | #3868, Cell Signaling Technology | 1:1000 for WB |
| ZO-1 | ab221547, Abcam | 1:100 for IF |
| CD31 | GB113151-100, Servicebio Technology | 1:300 for IF |
| CD63 | ab271286, Abcam | 1:50 for IF |
| TGF-β1 | GB13028, Servicebio Technology | 1:50 for IF |
| HA-AF594 | M10004M, Abmart | 1:200 for IF |
| Rab5 | #3547, Cell Signaling Technology | 1:200 for IF |
| Rab7 | ab126712, Abcam | 1:200 for IF |
| Golgin-97 | A21270, Invitrogen | 1:500 for IF |
| Purified anti-mouse CD16/32 | 93, Biolegend | 1:50 for flow cytometry |
| PE/Cyanine7 anti-mouse CD45 | S18009F, Biolegend | 1.25:100 for flow cytometry |
| APC anti-mouse CD31 | 390, Biolegend | 1.25:100 for flow cytometry |
| PE anti-mouse VE-cadherin | VECD1, Biolegend | 1:20 for flow cytometry |
| S100A4 | ab27957, Abcam | 1:50 for flow cytometry |
| Goat anti-mouse IgG HRP | M21001, Abmart | 1:5000 for WB |
| goat anti-Rabbit IgG HRP | M21002, Abmart | 1:5000 for WB |
| mouse anti-rabbit IgG HRP | M21006, Abmart | 1:1000 for WB |
| goat anti-rabbit IgG HRP | M21007, Abmart | 1:1000 for WB |
| goat anti-mouse IgG HRP | M21004, Abmart | 1:1000 for WB |
| goat anti-mouse IgG HRP | M21005, Abmart | 1:1000 for WB |
