# Supplementary figures and images for: The incorporation of acetylated LAP-TGF-β1 proteins into exosomes promotes TNBC cell dissemination in lung micro-metastasis
Source: Mol Cancer. 2024 Apr 25;23:82. doi: 10.1186/s12943-024-01995-z (PMC11044330; doi:10.1186/s12943-024-01995-z)

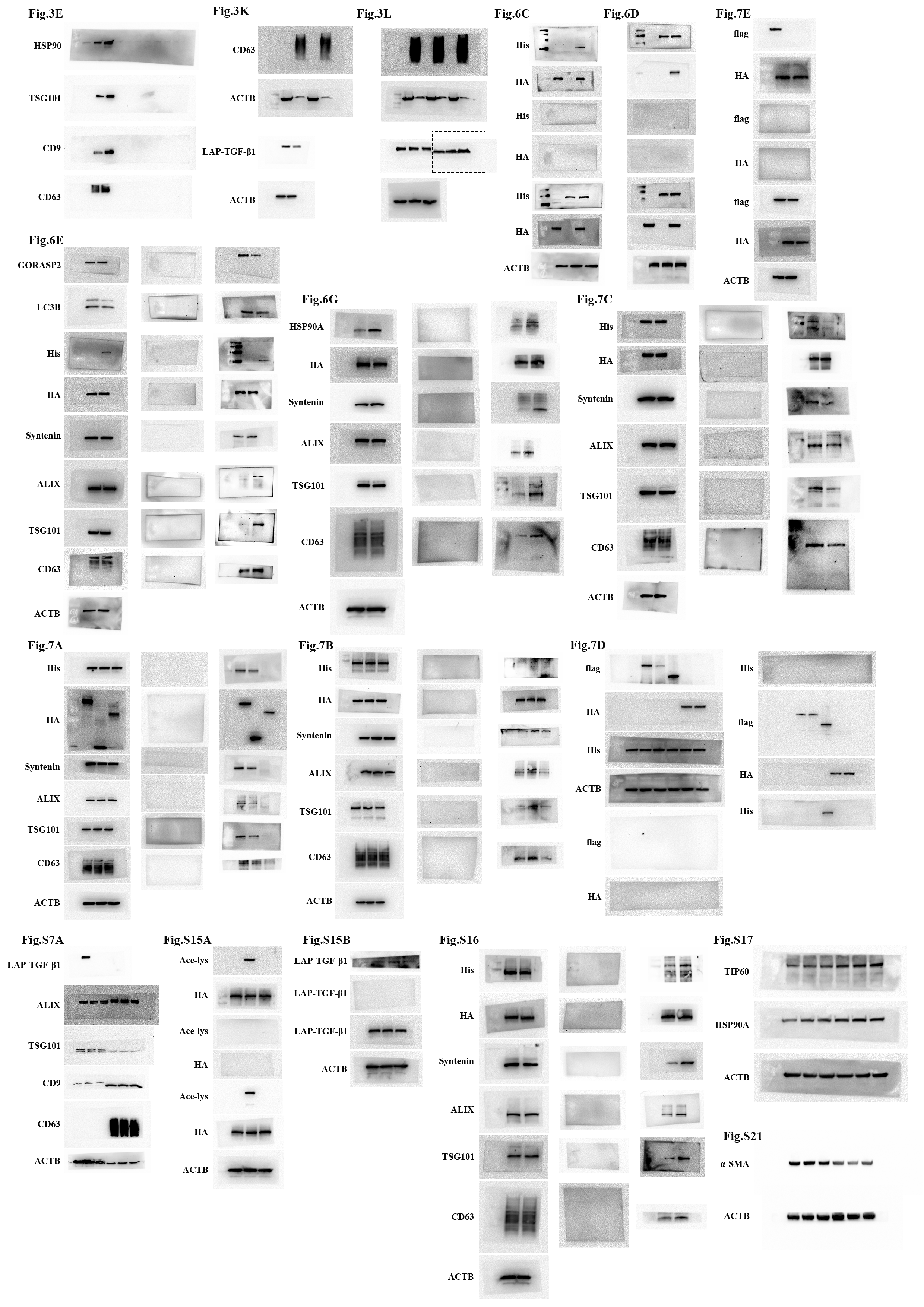

Supplement: Supplementary file 2 — Supplementary Material 2 [file 12943_2024_1995_MOESM2_ESM.tif]
